# Significance of supervision sampling in control of communicable respiratory disease simulated by a new model during different stages of the disease

**DOI:** 10.1038/s41598-025-86739-9

**Published:** 2025-01-30

**Authors:** Alphonse Houssou Hounye, Xiaogao Pan, Yuqi Zhao, Cong Cao, Jiaoju Wang, Abidi Mimi Venunye, Li Xiong, Xiangping Chai, Muzhou Hou

**Affiliations:** 1 General Surgery Department of Second Xiangya Hospital, Central South University Changsha, 139 Renmin Road, Changsha, Hunan 410011 China; 2 Department of Emergency Medicine, Second Xiangya Hospital, Central South University, Changsha, China; 3 Department of Gastroenterology, The Second Xiangya Hospital, Central South University, Changsha, Hunan China; 4 School of Mathematics and Statistics, Central South University, Changsha, 410083 China; 5 Emergency Medicine and Difficult Diseases Institute, Second Xiangya Hospital, Central South University, Changsha, 139 Renmin Road, Changsha, 410011 Hunan China

**Keywords:** SARS-CoV-2 transmission, Disease-free equilibrium, Transmission rate, SEI(A)R epidemic model, Preventive medicine, Applied mathematics

## Abstract

The coronavirus disease 2019 (COVID-19) interventions in interrupting transmission have paid heavy losses politically and economically. The Chinese government has replaced scaling up testing with monitoring focus groups and randomly supervising sampling, encouraging scientific research on the COVID-19 transmission curve to be confirmed by constructing epidemiological models, which include statistical models, computer simulations, mathematical illustrations of the pathogen and its effects, and several other methodologies. Although predicting and forecasting the propagation of COVID-19 are valuable, they nevertheless present an enormous challenge. This paper emphasis on pandemic simulation models by introduced respiratory-specific transmission to extend and complement the classical Susceptible-Exposed-(Asymptomatic)-Infected-Recovered SE(A)IR model to assess the significance of the COVID-19 transmission control features to provide an explanation of the rationale for the government policy. A novel epidemiological model is developed using mean-field theory. Utilizing the SE(A)IR extended framework, which is a suitable method for describing the progression of epidemics over actual or genuine landscapes, we have developed a novel model named SEIAPUFR. This model effectively detects the connections between various stages of infection. Subsequently, we formulated eight ordinary differential equations that precisely depict the population’s temporal development inside each segment. Furthermore, we calibrated the transmission and clearance rates by considering the impact of various control strategies on the epidemiological dynamics, which we used to project the future course of COVID-19. Based on these parameter values, our emphasis was on determining the criteria for stabilizing the disease-free equilibrium (DEF). We also developed model parameters that are appropriate for COVID-19 outbreaks, taking into account varied population sizes. Ultimately, we conducted simulations and predictions for other prominent cities in China, such as Wuhan, Shanghai, Guangzhou, and Shenzhen, that have recently been affected by the COVID-19 outbreak. By integrating different control measures, respiratory-specific modeling, and disease supervision sampling into an expanded SEI (A) R epidemic model, we found that supervision sampling can improve early warning of viral activity levels and superspreading events, and explained the significance of containments in controlling COVID-19 transmission and the rationality of policy by the influence of different containment measures on the transmission rate. These results indicate that the control measures during the pandemic interrupted the transmission chain mainly by inhibiting respiratory transmission, and the proportion of supervision sampling should be proportional to the transmission rate, especially only aimed at preventing a resurgence of SARS-CoV-2 transmission in low-prevalence areas. Furthermore, The incidence hazard of Males and Females was 1.39(1.23–1.58), and 1.43(1.26–1.63), respectively. Our investigation found that the ratio of peak sampling is directly related to the transmission rate, and both decrease when control measures are implemented. Consequently, the control measures during the pandemic interrupted the transmission chain mainly by inhibiting respiratory transmission. Reasonable and effective interventions during the early stage can flatten the transmission curve, which will slow the momentum of the outbreak to reduce medical pressure.

## Introduction

In the absence of global containment, the current COVID-19 pandemic still calls for modeling tools to assess disease features in order to avoid greater social, economic and political disruption^1,2^. A common goal is to capture viral spread patterns and predictably adopt available policies to interrupt transmission before the peak of infection, which may be a long-term strategy for future responses to COVID-19^3^. Until SARS-CoV-2 testing is restarted on a wide scale, a possible strategy would be to reflect epidemiologic trends in the overall population in real time via multistage sampling, as noted in our previous studies^4^. However, modeling efforts for multistage sampling would be difficult since the rates of transmission and clearance would change in response to control measures such as masks, quarantines, and vaccines, which is one of the common shortcomings of the classic SEI(A)R model^5^. To address these issues, disease-specific parameter drivers need to be modified and tested with different control measures imposed on the disease to see how pandemic transmission patterns change^6,7^.

The other aspect is that traditional epidemiologic models rarely capture well the spread differences between respiratory-specific and nonrespiratory infectious diseases^8^. For example, the classical SE(A)IR models were used for epidemiological prediction and control of the West Nile virus pandemic in North America in the 1990s^9^ and the SARS pandemic in Asia in 2003^10^, but were unable to directly distinguish the spreading characteristics of these two infectious diseases in the modeling logics. Based on these concerns, we introduced respiratory-specific transmission to extend and complement the classical SE(A)IR model to assess the influence of different control measures on COVID-19 transmission.

A new epidemiological model is constructed based on mean-field theory^11,12^. First, based on the SE(A)IR extended framework, a natural framework to describe epidemic dynamics unfolding over real or realistic landscapes, we built a new model called SEIAPUFR that identifies the relationships among different phases of infection. Then we established eight ordinary differential equations that describe the population’s evolution through time in each compartment. Second, we adjusted the rates of transmission and clearance based on the influence of different control measures on the epidemiologic process which we used to extrapolate the future trajectory of COVID-19. On the basis of these parameter settings, we focused on the stabilization conditions of disease-free equilibrium (DEF)^2^, and derived model parameters suitable for COVID-19 outbreaks according to different population sizes. Finally, we conduct simulations and forecasts for several first-tier cities in China that recently attacked COVID-19, including Wuhan, Shanghai, Guangzhou, and Shenzhen.

**Fig. 1:**
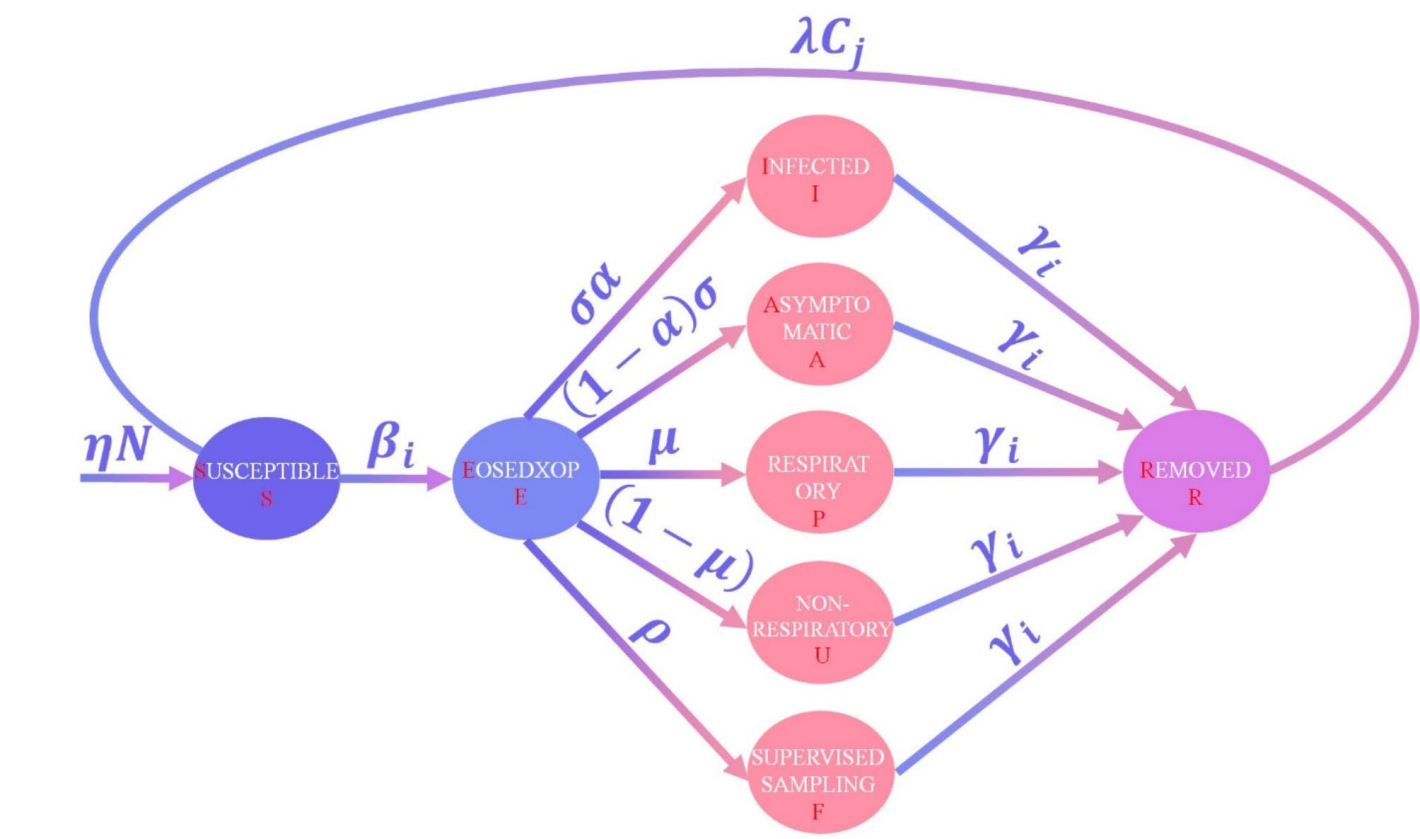
In the SEIAPUFR model, a graphical scheme represents the relationships among different phases of infection: S, susceptible (uninfected); E, exposed (those who have been polluted but are not yet infectious due to the incubation period); I, infected (asymptomatic or paucisymptomatic infected, undiscovered); A, diagnosed (asymptomatic infected, identified); P, respiratory specific model (symptomatic infected, detected); U, non-respiratory specific model; F, supervised sampling in the infected population (detected); R, healed (removed).

## Methods

### Modeling instructions

Here, we consider a large-scale metapopulation model describing a set of cities with baseline population Ni connected by human mobility; the whole population is subdivided into the COVID-19-relevant eight epidemiological compartments, which are: susceptible populations (Si, uninfected), exposed populations (Ei, contaminated but uninfected due to incubation period), infected populations (Ii, symptomatic or mildly symptomatic infection), asymptomatic population (Ai, asymptomatic infection), respiratory-specific model (Pi, respiratory-transmitted infection, e.g., droplets, aerosols), non-respiratory-specific population (Ui, non-respiratory-transmitted, e.g., contact, diet), supervised sampling of infected populations (Fi, detected), and recovered individuals (Ri) for each city i (show in Fig. 1).

1 $$\:\begin{array}{l}\left\{\begin{array}{l}\frac{dS\left(t\right)}{dt}=\eta\:N-\frac{{\beta\:}_{i}S\left(t\right)\left(E\left(t\right)+A\left(t\right)+P\left(t\right)+U\left(t\right)\right)}{N}-{\sum\:}_{j=1}^{4}{C}_{j}\lambda\:S\left(t\right)-\frac{g\tau\:S\left(t\right)}{N}\\\:\frac{dE\left(t\right)}{dt}=\frac{{\beta\:}_{i}S\left(t\right)\left(E\left(t\right)+A\left(t\right)+P\left(t\right)+U\left(t\right)\right)}{N}-\sigma\:E\left(t\right)\\\:\frac{dI\left(t\right)}{dt}=\sigma\:\alpha\:E\left(t\right)-{\gamma\:}_{i}I\left(t\right)\\\:\frac{dA\left(t\right)}{dt}=\sigma\:\left(1-\alpha\:\right)E\left(t\right)-{\gamma\:}_{i}A\left(t\right)\\\:\frac{dP\left(t\right)}{dt}=\mu\:E\left(t\right)-{\gamma\:}_{i}P\left(t\right)\\\:\mu\:=1-\text{exp}\left\{-\frac{\left(I\left(t\right)+A\left(t\right)+P\left(t\right)+U\left(t\right)\right)qpt}{Q}\left[1-\text{exp}\left(-\frac{Qt}{V}\right)\right]\right\}\\\:\frac{dU\left(t\right)}{dt}=\left(1-\mu\:\right)E\left(t\right)-{\gamma\:}_{i}U\left(t\right)\\\:\frac{dF\left(t\right)}{dt}={\vartheta\:}_{g}{N}^{\text{*}}\text{log}\left(\frac{k}{F\left(t\right)}\right)+\rho\:E\left(t\right)-{\gamma\:}_{i}F\left(t\right)\\\:{N}^{*}=\frac{({N}^{+}-((1-{s}_{PEC})\left)N\right)}{{S}_{en}+{S}_{pec}-1}\\\:\frac{dR\left(t\right)}{dt}={\gamma\:}_{i}\left(I\left(t\right)+A\left(t\right)+P\left(t\right)+U\left(t\right)\right)\end{array}\right.\end{array}$$

All of the parameters evaluated are positive integers indicated by Greek letters. The interconnections between different phases of infection are illustrated in Fig. 1. The following are the parameters:

Parameter η is the inflow rate (including birth and immigration). For each period i, the transmission rate βi(the risk of disease transmission in a single encounter multiplied by the average number of contacts per person) occurs when a susceptible subject comes into touch with an exposed, infected, asymptomatic, respiratory-specific model or non-respiratory specific. We assume that susceptible people get vaccinated at a constant rate per capita Cj(0 ≤ Cj < 1, j ∈ {0, 1, 2, 3, 4}) (i.e., vaccine coverage rate). The vaccination effectiveness is λ(0 ≤ λ ≤ 1). When λ = 0, the vaccination has no effect. In contrast, when λ = 1, the vaccine is completely effective. g is the number of loci that were associated with SARS-CoV-2 infection and disease severity. $\:\tau\:$ is the probability that those loci affect the general susceptibility population. These parameters can be changed since they impact various things, including government epidemic prevention efforts, quarantine, and immunization. The danger of infection from threatening people treated in adequate ICUs is low.

α and σ capture the incubation rate of detection relative to asymptomatic and symptomatic cases, respectively. These parameters reflect the level of attention on the disease and the number of tests performed over the population: they can be increased by enforcing a massive contact tracing. Moreover, these parameters are also the rate at which the exposed and asymptomatic population becomes infected. µ is the probability of infection for a susceptible population, which is the Wells-Riley mathematical equation developed by^13^) to express the transmission potential of infection as a function of the fraction of inhaled air that has been previously exhaled by someone in the building (i.e., rebreathed fraction) using CO2 concentration as a marker for exhaled-breath exposure. An infected person’s quantum generation rate is q(quanta/h). The breathing rate per individual is p(m3/h). The entire exposure time is t(h). V is the volume of the ventilated space (m3), and Q is the exterior air supply rate (m3/h). Based on the total CO2 level in the indoor air contributed from human origin and outdoor air supply, and person-to-person transmission of infectious diseases through recirculation air in the ventilation airspace, the outdoor air supply rate (Q) can be expressed as functions of the fraction of indoor that is exhaled breath (f ), people in the ventilation airspace (n), and breathing rate per person (p), as Q = np/f.

ρ is the probability rate at which the exposed become infected among the whole population. N^∗^ is the estimated number of SARS-CoV-2 infections correcting for imperfect test accuracy^14^). ϑg the parameters for the growth rate in the model, k is the maximum values of the model attained as t goes to infinity, N^+^ is the sum of the number of confirmed cases (tested positive), Sensitivity(Sen) < 1, or Specificity (Spec) < 1. These parameters are test-dependent but may be partially increased by improving the test efficacy and acquiring immunity against the virus.

The rate of infected subjects being removed is denoted by γi; it may differ significantly if an appropriate treatment for the disease is known and adopted for diagnosed patients, such as the perfect test and the vaccine efficacy at each specific period i, but otherwise, they are likely comparable. This value can be raised due to better therapies and viral immunity.

### Description of modeling approaches

We consider the probability rate of becoming susceptible again, after having recovered from the infection, in the model because of the vaccine efficacy and imperfect correction of the test among the entire population, which appears not to be negligible since the epidemic can be transmitted without symptoms and posed a crucial problem for knowing the real infectious time frame and potential exposures^15^). According to several studies, asymptomatic and presymptomatic COVID-19 infections are equally transmissible in the early days of the illness^16^). Although some data suggest that SARS-CoV-2 has resurfaced^17^), the presence of viral RNA in respiratory samples might indicate persistence rather than return. More extensive studies have found that 30–70% of persons who test positive have little or no symptoms^18^) and that asymptomatic and pre-symptomatic people can transmit the pandemic^19^). The literature is still looking at the subacute and long-term effects of COVID-19, which can affect multiple organ systems^20^). Early evidence suggests that SARS-CoV-2 infection causes long-term effects, including tiredness, dyspnea, chest discomfort, cognitive problems, arthralgia, and declining quality of life^21^). These consequences might be caused by cellular damage, an inflammatory cytokine-producing innate immune response, or a pro-coagulant state caused by SARS-CoV-2 infection^22^). Previous coronavirus infections, such as the SARS epidemic in 2003 and the Middle East respiratory sickness (MERS) outbreak in 2012, have left survivors with a similar set of symptoms, raising concerns about COVID-19’s clinically significant consequences^23^).

SARS-CoV-2 has been found in asymptomatic people^24^), suggesting that subclinical active infection is a major factor in this epidemic. COVID-19 is often diagnosed by a reverse transcription polymerase chain reaction (RT-PCR) viral RNA test, which appears to be sensitive to the assay technique as well as the time of specimen collection, transit, and storage^24^). As a result, many infected persons probably remained undiscovered because they were subclinical or asymptomatic. We were particularly interested in predicting the effect of the respiratory-specific model, as well as supervision sampling, where temporary immunity is still likely in place, and the possibility of reinfection would have a significant impact on the total number of susceptible individuals, resulting in significant differences in the evolution of the pandemic curves we examined. We incorporate the effect of engineering control measures like increased air exchange and air filtration rates with personal masking, as well as public health interventions like vaccination, isolation, social distancing mandates, transmission dampening through the use of face masks, and contact tracing, on containing the spread of COVID-19 indoor and outdoor airborne infections to back up this claim. We utilize these findings to learn about epidemic progression and thereby model the initial wave of transmission using a deterministic SEIR compartmental framework^25–27^). This process-based knowledge of how non-pharmaceutical interventions (NPIs) alter epidemiological processes is utilized to predict COVID-19’s future trajectory and how various existing NPIs may affect it. Furthermore, when the potential of reinfection is given, the numerical simulation results of the model with varied population sizes show that the development is not similar. The main difference is that the restored population diminishes with time. As a result, while we cannot rule out the possibility that adaptive immunity to SARS-CoV-2 may not give long-term protection, we can safely assume that the risk of reinfection is not insignificant within the scope of our model.

Our approach distinguishes between non-diagnosed people who spread the illness more because they are not isolated and diagnosed people who spread the disease considerably less because they are correctly isolated and follow tight procedures, whether in the hospital or at home. Our model also takes into consideration the differential between the respiratory and non-respiratory populations, as well as the supervisory sampling on a single day throughout the epidemic. SARS-CoV-2 transmission from person to person in the home has been documented in China^28,29^). Although infection of COVID-19-positive persons’ household members is feasible, the disease incidence is difficult to determine now. Separating sick persons in designated quarantine centers is the only method to eliminate this risk^30^). Furthermore, our model considers vaccination efficiency and the total dosage received by the population throughout the pandemic.

Our model does not account for a possible latency between exposure to the virus and onset of infectiousness because there is mounting evidence that an infected individual can transmit the virus at an early, preclinical stage of the disease, based on epidemiological investigation of COVID-19 clusters^31,32^). Furthermore, recent studies have found that the median serial interval values for COVID-19 are near or less than the median incubation time^33^), indicating that the illness can be transmitted before symptoms appear. As a result, we did not think it was essential to introduce more stages: even if they are asymptomatic, those who have been exposed to the virus can still transmit it; thus, they fall in between the infected and diagnosed phases.

Finally, the SEIAPUFR model is a mean-field model that captures the average influence of occurrences affecting the whole population. Social mixing tendencies are averaged throughout the population and integrated into our parameters. Our model, on the other hand, is fully adaptable and suitable for incorporating features such as a distinction between respiratory and non-respiratory population groups and the importance of adjusting COVID-19 infection estimates for testing practices and diagnostic accuracy during periods of low testing rates. Another potential future development is to extend the model to predict the concurrent evolution of other diseases that, due to the epidemic emergency, may be overestimated, underestimated, or not treated appropriately because the healthcare system is overburdened, resulting in an increased number of ’collateral’ deaths not directly linked to the virus.

### Analysis of the mathematical model

The SEIAPUFR model (1) is an eight-differential- equation bilinear system. The system is positive: if all state variables start with non-negative values at 0, they will take non-negative values for t ≥ 0.

When the total population size N (t) varies, it is frequently required to evaluate the proportions of people in eight epidemiological classes.

$$\:s=\frac{S}{N},e=\frac{E}{N},i=\frac{I}{N},a=\frac{A}{N},p=\frac{P}{N},u=\frac{U}{N},f=\frac{F}{N},r=\frac{R}{N}$$

It is easy to check that s, e, i, a, p, u, f, and r fulfill the following set of differential equations:

2 $$\:\begin{array}{l}\left\{\begin{array}{l}\dot{s}=\eta\:-{\beta\:}_{i}s\left(e+a+p+u\right)-{\sum\:}_{j=1}^{4}{C}_{J}{\lambda\:}_{s}-g\tau\:sN,\\\:\dot{e}={\beta\:}_{i}s\left(e+a+p+u\right)-\sigma\:e,\\\:\dot{i}=\sigma\:\alpha\:e-{\gamma\:}_{i}i,\\\:\dot{a}=\sigma\:\left(1-\alpha\:\right)e-{\gamma\:}_{i}a,\\\:\dot{p}=\mu\:e-{\gamma\:}_{i}p,\\\:\dot{u}=\left(1-\mu\:\right)e-{\gamma\:}_{i}u,\\\:\dot{f}=\vartheta\:g{N}^{*}\text{log}\left(\frac{k}{f}\right)+\rho\:e-{\gamma\:}_{i}f,\\\:\dot{r}={\gamma\:}_{i}\left(i+a+p+u\right)\end{array}\right.\end{array}$$

The system is compartmental and exhibits the masse conservation property: $\:\dot{s}+\dot{e}+\dot{i}+\dot{a}+\dot{p}+\dot{u}+\dot{f}+\dot{r}=0$, they are implying that the total population remains constant. Because the variables represent population fractions, they are restricted to $\:s+e+i+a+p+u+f\:+r\:=\:1$, where 1 represents the whole population.

The variables r are absent from the first seven equations of (2). First, we will look at the simplified system.

3 $$\:\left\{\begin{array}{l}\dot{s}=\eta\:-{\beta\:}_{i}s\left(e+a+p+u\right)-{\sum\:}_{j=1}^{4}{C}_{J}{\lambda\:}_{s}-g\tau\:sN,\\\:\dot{e}={\beta\:}_{i}s\left(e+a+p+u\right)-\sigma\:e,\\\:\dot{i}=\sigma\:\alpha\:e-{\gamma\:}_{i}i,\\\:\dot{a}=\sigma\:\left(1-\alpha\:\right)e-{\gamma\:}_{i}a,\\\:\dot{p}=\mu\:e-{\gamma\:}_{i}p,\\\:\dot{u}=\left(1-\mu\:\right)e-{\gamma\:}_{i}u\\\:\dot{f}=\vartheta\:g{N}^{*}\text{log}\left(\frac{k}{f}\right)+\rho\:e-{\gamma\:}_{i}f\end{array}\right.$$

and calculate r using $\:r=1-s-e-i-a-p-u-f$ or $\:\dot{r}={\gamma\:}_{i}(i+a+p+u)$. The equation’s feasible region (3) is

4 $$\:\begin{array}{l}\varOmega = \left\{\left(s,e,i,a,p,u,f\right)\in\:{\mathfrak{R}}_{+}^{7}|0\le\:s+e+i+a+p+u+f\le\:1\right\}\end{array}$$

which can be shown to be positivity invariant (i.e., given non-negative beginning values in Ω, have non-negative components and stay in Ω for t ≥ 0) and universally attractive in $\:{\mathcal{R}}_{+}^{7}$with respect to (3). As a result, we concentrate on the dynamics of (3) in Ω. ∂Ω and Ω◦, respectively, represent the boundary and inside of Ω.

### The basic reproduction number and DFE (Disease-Free Equilibrium)

#### The basic reproduction number

The basic reproduction number, as an indicator of disease transmission within a community, is crucial for understanding and managing a continuing pandemic. It is defined as the mean number of infections produced by one contaminated person during their contagious stage in a completely healthy community. Employing the next generation matrix methodology for our framework (1), the basic reproduction number may be determined by analyzing the generation matrices F and V, which represent the Jacobian matrices related to the emergence of new viruses and the net transition rate from the respective areas, accordingly

$$\:F=\left[\begin{array}{ccccc}\frac{{\beta\:}_{i}S}{N}&\:0&\:0&\:0&\:0\\\:0&\:0&\:0&\:0&\:0\\\:0&\:0&\:0&\:0&\:0\\\:0&\:0&\:0&\:0&\:0\\\:0&\:0&\:0&\:0&\:0\end{array}\right],\:\text{a}\text{n}\text{d}\:V=\left[\begin{array}{ccccc}\sigma\:&\:0&\:0&\:0&\:0\\\:-\sigma\:&\:{\gamma\:}_{i}&\:0&\:0&\:0\\\:-\sigma\:\left(1-\alpha\:\right)&\:0&\:{\gamma\:}_{i}&\:0&\:0\\\:0&\:0&\:-\mu\:&\:{\gamma\:}_{i}&\:0\\\:0&\:0&\:-\left(1-\mu\:\right)&\:0&\:{\gamma\:}_{i}\end{array}\right]\:.$$

The basic reproduction number is given by the spectral radius of the product matrix $\:F{V}^{-1}$.

$$\:{R}_{0}=\frac{{\beta\:}_{i}S}{{\gamma\:}_{i}N}.$$

#### DFE (Disease-Free Equilibrium)

There are two particular concerns when addressing a disease’s eradication in a population of varied sizes. The more challenging method of eradicating the pandemic (COVID-19) requires that the overall number of infected people be $\:E\left(t\right)+I\left(t\right)+A\left(t\right)+P\left(t\right)+U\left(t\right)+F\left(t\right) \to 0$, whereas the weaker method requires that the proportion sum be $\:e\left(t\right)+i\left(t\right)+a\left(t\right)+p\left(t\right)+p\left(t\right)+f\left(t\right) \to 0$ (confer^34^ for more details). We are motivated to look for conditions that will allow the DFE to exist and be stable for$\:\:\:{G}_{0}(\frac{\eta\:}{{\sum\:}_{j=1}^{4}{C}_{j}\lambda\:+g\tau\:sN},0,\text{0,0},\text{0,0},{f}_{0})$ and inherent equilibrium point $\:{G}^{*}({s}^{*},{e}^{*},{i}^{*},{a}^{*},{p}^{*},{u}^{*},{f}^{*})$. Clearly, the DFE of (3) is $\:{G}_{0}(\frac{\eta\:}{{\sum\:}_{j=1}^{4}{C}_{j}\lambda\:s-g\tau\:sN},\text{0,0},\text{0,0},0,{f}_{0})\in\:{\Omega\:}$ which exists for all positive parameters. At each point $\:G(s,\:e,\:i,\:a,\:p,\:u,\:f)$ the Jacobian matrix of (3) takes the form:

$$\:J\left(G\right)=\left(\begin{array}{lllllll}\eta\:-{\beta\:}_{i}(e+a+p+u)-\sum\:_{j=1}^{4}{C}_{j}\lambda\:-g\tau\:N&\:-{\beta\:}_{i}s&\:0&\:-{\beta\:}_{i}s&\:-{\beta\:}_{i}s&\:-{\beta\:}_{i}s&\:0\:\\\:{\beta\:}_{i}(e+a+p+u)&\:{\beta\:}_{i}s-\sigma\:&\:0&\:{\beta\:}_{i}s&\:{\beta\:}_{i}s&\:{\beta\:}_{i}s&\:0\\\:0&\:\alpha\:\sigma\:&\:-{\gamma\:}_{i}&\:0&\:0&\:0&\:0\\\:0&\:\alpha\:(1-\sigma\:)&\:0&\:-{\gamma\:}_{i}&\:0&\:0&\:0\\\:0&\:\mu\:&\:0&\:0&\:-{\gamma\:}_{i}&\:0&\:0\\\:0&\:1-\mu\:&\:0&\:0&\:0&\:-{\gamma\:}_{i}&\:0\\\:0&\:\rho\:&\:0&\:0&\:0&\:0&\:-\xi\:\end{array}\right)$$

Where $$\:\xi\:=\frac{{\vartheta\:}_{g}{N}^{\text{*}}}{f}\left(\vartheta\:g{N}^{\text{*}}\text{l}\text{o}\text{g}\left(\frac{k}{f}\right)+\rho\:e-{\gamma\:}_{i}f\right)+{\gamma\:}_{i}$$.

We derive the characteristic equation of J(G) at G = G0 to examine the stability of DFE:

5 $$\begin{aligned} & \left(\bar \lambda -{\gamma\:}_{i}\right)\left(\xi\:-\bar \lambda\right)\left(\eta\:-\sum\:_{j=1}^{4}\:\:{C}_{j}\lambda\:-g\tau\:sN-\bar \lambda\right){\left({\gamma\:}_{i}+\bar \lambda \right)}^{2} \\ & \quad \left({\bar \lambda}^{2}+\left({\gamma\:}_{i}-{\beta\:}_{i}\frac{\eta\:}{\sum\:_{j=1}^{4}\:\:{C}_{j}\lambda\:+g\tau\:sN}\right) \bar \lambda+{\beta\:}_{i}\frac{\eta\:}{\sum\:_{j=1}^{4}\:\:{C}_{j}\lambda\:+g\tau\:sN} -\left({\beta\:}_{i}\frac{\eta\:}{\sum\:_{j=1}^{4}\:\:{C}_{j}\lambda\:+g\tau\:sN}\left({\gamma\:}_{i}+\sigma\:\left(1-\alpha\:\right)\right)\right)\right)=0 \end{aligned}$$

The stability of G0 is equal to all (5) eigenvalues having a negative real portion, which is guaranteed by

6 $$\:\begin{array}{l}{R}_{0}=\frac{{\gamma\:}_{i}+\sigma\:\left(1-\alpha\:\right)}{{\gamma\:}_{i}^{2}\xi\:\left(\eta\:-\sum\:_{j=1}^{4}\:\:{C}_{j}\lambda\:-g\tau\:sN\right)}\end{array}$$

The epidemiological threshold parameter R_0_ is used here. As a result, if R_0_ < 1, the DFE is locally asymptotically stable.

7 $$\:\begin{array}{l}{\widehat{R}}_{0}=\frac{\sigma\:\left(1-\alpha\:\right)}{{\gamma\:}_{i}^{2}\xi\:\left(\eta\:-\sum\:_{j=1}^{4}\:\:{C}_{j}\lambda\:-g\tau\:sN\right)}\end{array}$$

It finds that although $\:{\widehat{R}}_{0}$< 1 ensures $\:{R}_{0}$ < 1, the opposite is not true.

##### Theorem 1

If < 1, of (3) is globally asymptotically stable in Ω; if R0 > 1, it is unstable. Except for those starting on the invariant s- and f-axes, which approach G0 along these axes, the solution of (3) starting sufficiently near G0 in Ω moves farther away from G0.

**Proof of Theorem** **1**. We define an appropriate Lyapunov function $\:{L}_{func}=\sigma\:\alpha\:e+\sigma\:i+\mu\:a+\sigma\:\left(1-\alpha\:\right)p+\rho\:u+\left(1-\mu\:\right){f}^{2}$, to verify the global stability of G0. When $\:{L}_{func}$is differentiated along (3), $\:{L}_{\text{func}}^{{\prime\:}}\:$is obtained by formulas below

8 $$\:\begin{aligned}{L}_{\text{func}}^{{\prime\:}}& =\sigma\:\alpha\:\stackrel{\prime }{e}+\sigma\:\stackrel{\prime }{i}+\mu\:\stackrel{\prime }{a}+\sigma\:\left(1-\alpha\:\right)\stackrel{\prime }{p}+\rho\:\stackrel{\prime }{u}+2\left(1-\mu\:\right)f\stackrel{\prime }{f}\\\:& =\sigma\:\alpha\:{\beta\:}_{i}s\left(e+a+p+u\right)-\sigma\:\alpha\:e+{\sigma\:}^{2}\alpha\:e-\sigma\:{\gamma\:}_{i}i\\\:& \quad +\mu\:\sigma\:\left(1-\alpha\:\right)e-\mu\:\gamma\:ia+\sigma\:\left(1-\alpha\:\right)\mu\:e-{\gamma\:}_{i}\sigma\:\left(1-\alpha\:\right)p\\\:& \quad+\rho\:\left(1-\mu\:\right)e-{\gamma\:}_{i}\rho\:u+2\left(1-\mu\:\right){\vartheta\:}_{g}{N}^{\text{*}}\text{log}\left(\frac{k}{f}\right)f+2\left(1-\mu\:\right)\rho\:fe-2{\gamma\:}_{i}\left(1-\mu\:\right){f}^{2}\\\:& =e\left[\sigma\:\alpha\:{\beta\:}_{i}s-\sigma\:\alpha\:-{\sigma\:}^{2}\alpha\:-\mu\:\sigma\:\left(1-\alpha\:\right)+\sigma\:\left(1-\alpha\:\right)\mu\:+\rho\:\left(1+2f\right)\left(1-\mu\:\right)\right]-\sigma\:{\gamma\:}_{i}i\\\:& \quad+a\left[\sigma\:\alpha\:{\beta\:}_{i}-\mu\:{\gamma\:}_{i}\right]+p\left[\sigma\:\alpha\:{\beta\:}_{i}s-{\gamma\:}_{i}\sigma\:\left(1-\alpha\:\right)\right]+u\left[\sigma\:\alpha\:{\beta\:}_{i}s-{\gamma\:}_{i}\rho\:\right]\\\:& \quad+2\left[\left(1-\mu\:\right){\vartheta\:}_{i}{N}^{\text{*}}\text{flog}\left(\frac{k}{f}\right)-{\gamma\:}_{i}\left(1-\mu\:\right)f\right]f \end{aligned}$$

When R0 ≤ 1, the greatest value of (8) in Ω is attained at the extremal points: M1(0, 0, 0, 0, 0, 0, 0); M2(1, 0, 0, 0, 0, 0, 0); M3(0, 1, 0, 0, 0, 0, 0); M4(0, 0, 1, 0, 0, 0, 0); M5(0, 0, 0, 1, 0, 0, 0); M6(0, 0, 0, 0, 1, 0, 0); M7(0, 0, 0, 0, 0, 1, 0); M8(0, 0, 0, 0, 0, 0, 1). (8) becomes an equality $\:{L}_{\text{func}}^{{\prime\:}}=0$ ), if s = 0, e = 0, i = 0, a = 0, *p* = 0, u = 0, f = 0 or $\:{\widehat{R}}_{0}=1$. The singleton {G0} is the greatest invariant set in $\:\left\{\right(s,e,i,a,p,u,f)\in\:{\Omega\:}|{L}_{func}^{{\prime\:}}=0\}$. The DFE {G0} is globally asymptotically stable when $\:{\widehat{R}}_{0}$ ≤ 1, according to LaSalle’s Invariance Principal^35^), Chap. 2,Theorem, 6.4.

$$\:\text{W}\text{e}\:\text{d}\text{e}\text{f}\text{i}\text{n}\text{e}\:{L}_{\text{secfunc}\text{\:}}=\sigma\:\alpha\:e+\sigma\:i+\mu\:a+\sigma\:(1-\alpha\:)p+\rho\:u+(1-\mu\:){f}^{2},\:\text{i}\text{f}\:{R}_{0}>1.$$

9 $$\:\begin{aligned}{L}_{\text{secfunc}\text{\:}}^{{\prime\:}}& =\sigma\:\alpha\:\stackrel{\prime }{e}+\sigma\:i+\mu\:\stackrel{\prime }{a}+\sigma\:\left(1-\alpha\:\right)\stackrel{\prime }{p}+\rho\:\stackrel{\prime }{u}+2\left(1-\mu\:\right)f\stackrel{\prime }{f}\\\:& =\sigma\:\alpha\:{\beta\:}_{i}s\left(e+a+p+u\right)-\sigma\:\alpha\:e+{\sigma\:}^{2}\alpha\:e-\sigma\:{\gamma\:}_{i}i\\\:& \quad +\mu\:\sigma\:\left(1-\alpha\:\right)e-\mu\:\gamma\:ia+\sigma\:\left(1-\alpha\:\right)\mu\:e-{\gamma\:}_{i}\sigma\:\left(1-\alpha\:\right)p\\\:&\:\:+\rho\:\left(1-\mu\:\right)e-{\gamma\:}_{i}\rho\:u+2\left(1-\mu\:\right){\vartheta\:}_{g}{N}^{\text{*}}\text{log}\left(\frac{k}{f}\right)f+2\left(1-\mu\:\right)\rho\:fe-2{\gamma\:}_{i}\left(1-\mu\:\right){f}^{2}\\\:& =e\left[\sigma\:\alpha\:{\beta\:}_{i}s-\sigma\:\alpha\:-{\sigma\:}^{2}\alpha\:-\mu\:\sigma\:\left(1-\alpha\:\right)+\sigma\:\left(1-\alpha\:\right)\mu\:+\rho\:\left(1+2f\right)\left(1-\mu\:\right)\right]-\sigma\:{\gamma\:}_{i}i\\\:& \quad +a\left[\sigma\:\alpha\:{\beta\:}_{i}-\mu\:{\gamma\:}_{i}\right]+p\left[\sigma\:\alpha\:{\beta\:}_{i}s-{\gamma\:}_{i}\sigma\:\left(1-\alpha\:\right)\right]+u\left[\sigma\:\alpha\:{\beta\:}_{i}s-{\gamma\:}_{i}\rho\:\right]\\\:& \quad +2\left[\left(1-\mu\:\right){\vartheta\:}_{i}{N}^{\text{*}}\text{flog}\left(\frac{k}{f}\right)-{\gamma\:}_{i}\left(1-\mu\:\right)f\right]f\end{aligned}$$

when $\:e=i=a=u=f=0,{L}_{\text{secfunc\:}}^{{\prime\:}}>0$ for $\:s$, which is sufficiently close to $\:\frac{\eta\:}{{\sum\:}_{j=1}^{4}\:{C}_{j}\lambda\:+g\tau\:sN}$. Except for those on the invariant s- and f-axes, solutions staring sufficiently near to G0 depart a neighborhood of G0 when (3) reduces to $\:\stackrel{\prime }{s}=\eta\:-{\sum\:}_{j=1}^{4}\:{C}_{j}\lambda\:s-g\tau\:sN$ and therefore $\:s\left(t\right)\to\:\eta\:-{\sum\:}_{j=1}^{4}\:{C}_{j}\lambda\:-g\tau\:sN$ as $\:t\to\:{\infty\:}$.

It should be noted that eigenvalue analysis of J(G) may also verify the unstable nature of DFE G0 when R0 > 1. The threshold parameters R0 and $\:{\widehat{R}}_{0}$ determine whether the infected fraction (e(t), i(t), a(t), p(t), u(t), f (t)) vanishes locally or globally in time. Reducing R0 to values less than unity can effectively ”eradicate” the epidemic (COVID-19). When $\:{\widehat{R}}_{0}$ is changed to a value less than or equal to unity, the pandemic can be ”eradicated” even if it is enormous. R0 and $\:{\widehat{R}}_{0}$ may be understood epidemiologically as disease transmission via contacts becoming stronger as both susceptible inflow and exposed ones grow. Both R0 and $\:{\widehat{R}}_{0}$ are discovered to be decreasing functions of λ. If $\:\lambda\:<\frac{\eta\:}{1+\eta\:}$ R0 rises with Cj and falls with Cj if implying that the high vaccination rate combined with low vaccine efficacy will likely make the pandemic ”persistent.” As the immunization rate λ rises, $\:{\widehat{R}}_{0}$decreases.

### Epidemic outbreak function based on our model

Given the multitude of variables influencing the values of $\:\beta\:$ and $\:\gamma\:$, such as government actions for epidemic prevention, quarantine protocols, and vaccination efforts, it is not justifiable to treat $\:\beta\:$ and $\:\gamma\:$ as fixed constants. Furthermore, an alternative widely-used approach involves using a function to estimate the values of $\:\beta\:$ and $\:\gamma\:$, individually. This technique ensures the continuity of the values of $\:\beta\:$ and $\:\gamma\:$, resulting in frequent variations. Hence, this work integrates these two concepts, supposing that the values of $\:\beta\:$ and $\:\gamma\:$ remain constant inside a certain time frame, and are updated upon entering the subsequent period.

In a pandemic, the change rate of infected populations can indicate whether the disease is breaking out or fading. E, I, A, P, U, and F reflect the number of infections in the SEIAPUFR model(1). Those in E are still in incubation, but those in I, A, P, U, and F have already been infected. When the sum of E, I, A, P, U, and F change rates is more than 0, we can conclude that the disease is still spreading, that is

10 $$\:\begin{array}{l}\frac{d}{dt}\left(E+I+A+P+U+F\right)>0\end{array}$$

Which implies

11 $$\:\begin{array}{l}\frac{dE}{dt}+\frac{dI}{dt}+\frac{dA}{dt}+\frac{dP}{dt}+\frac{dU}{dt}+\frac{dF}{dt}>0\end{array}$$

By the SEIAPUFR model (1), we have

12 $$\:\begin{array}{l}\begin{array}{rr}&\:\frac{{\beta\:}_{i}S\left(t\right)\left(E\left(t\right)+A\left(t\right)+P\left(t\right)+U\left(t\right)\right)}{N}-\sigma\:E\left(t\right)+\sigma\:\alpha\:E\left(t\right)-{\gamma\:}_{i}I\left(t\right)\\\:&\:\:+\sigma\:\left(1-\alpha\:\right)E\left(t\right)-{\gamma\:}_{i}A\left(t\right)+\mu\:E\left(t\right)-{\gamma\:}_{i}P\left(t\right)+\left(1-\mu\:\right)E\left(t\right)-{\gamma\:}_{i}U\left(t\right)\\\:&\:\:+{\vartheta\:}_{g}{N}^{\text{*}}\text{log}\left(\frac{k}{F\left(t\right)}\right)+\rho\:E\left(t\right)-{\gamma\:}_{i}F\left(t\right)>0\end{array}\end{array}$$

It follows that

13 $$\:\begin{array}{l}\begin{array}{rr}&\:\frac{{\beta\:}_{i}S\left(t\right)\left(E\left(t\right)+A\left(t\right)+P\left(t\right)+U\left(t\right)\right)}{N}+\left(1+\rho\:\right)E\left(t\right)-{\gamma\:}_{i}I\left(t\right)\\\:&\:\:-{\gamma\:}_{i}A\left(t\right)-{\gamma\:}_{i}P\left(t\right)-{\gamma\:}_{i}U\left(t\right)+{\vartheta\:}_{g}{N}^{\text{*}}\text{log}\left(\frac{k}{F\left(t\right)}\right)-{\gamma\:}_{i}F\left(t\right)>0\end{array}\end{array}$$

$$\:\text{S}\text{u}\text{p}\text{p}\text{o}\text{s}\text{e}\:\text{t}\text{h}\text{a}\text{t}\:\text{t}\text{h}\text{e}\:\text{e}\text{x}\text{p}\text{r}\text{e}\text{s}\text{s}\text{i}\text{o}\text{n}$$ $$\:{\gamma\:}_{i}\left[-\frac{(1+\rho\:)}{{\gamma\:}_{i}}E\left(t\right)+I\left(t\right)+A\left(t\right)+P\left(t\right)+U\left(t\right)+F\left(t\right)-\frac{{\vartheta\:}_{g}{N}^{\text{*}}}{{\gamma\:}_{i}}\text{l}\text{o}\text{g}\left(\frac{k}{F\left(t\right)}\right)\right]>0$$

then we have,

14 $$\:\begin{array}{l}\frac{{\beta\:}_{i}S\left(t\right)\left(E\left(t\right)+A\left(t\right)+P\left(t\right)+U\left(t\right)\right)}{N{\gamma\:}_{i}\left[-\frac{\left(1+\rho\:\right)}{{\gamma\:}_{i}}E\left(t\right)+I\left(t\right)+A\left(t\right)+P\left(t\right)+U\left(t\right)+F\left(t\right)-\frac{{\vartheta\:}_{g}{N}^{\text{*}}}{{\gamma\:}_{i}}\text{log}\left(\frac{k}{F\left(t\right)}\right)\right]}>1\end{array}$$

The virus is disappearing when the rate of change of $\:E\left(t\right)+I\left(t\right)+A\left(t\right)+P\left(t\right)+U\left(t\right)+F\left(t\right)<0$. Using the above method, we have that

15 $$\:\begin{array}{l}\frac{{\beta\:}_{i}S\left(t\right)\left(E\left(t\right)+A\left(t\right)+P\left(t\right)+U\left(t\right)\right)}{N{\gamma\:}_{i}\left[-\frac{\left(1+\rho\:\right)}{{\gamma\:}_{i}}E\left(t\right)+I\left(t\right)+A\left(t\right)+P\left(t\right)+U\left(t\right)+F\left(t\right)-\frac{{\vartheta\:}_{g}{N}^{\text{*}}}{{\gamma\:}_{i}}\text{log}\left(\frac{k}{F\left(t\right)}\right)\right]}<1\end{array}$$

We know that the virus is under control and can coexist with people for a long time when the rate of change of $\:E\left(t\right)+I\left(t\right)+A\left(t\right)+P\left(t\right)+U\left(t\right)+F\left(t\right)=0$.

As a result, we have

16 $$\:\begin{array}{l}\frac{{\beta\:}_{i}S\left(t\right)\left(E\left(t\right)+A\left(t\right)+P\left(t\right)+U\left(t\right)\right)}{N{\gamma\:}_{i}\left[-\frac{\left(1+\rho\:\right)}{{\gamma\:}_{i}}E\left(t\right)+I\left(t\right)+A\left(t\right)+P\left(t\right)+U\left(t\right)+F\left(t\right)-\frac{{\vartheta\:}_{g}{N}^{\text{*}}}{{\gamma\:}_{i}}\text{log}\left(\frac{k}{F\left(t\right)}\right)\right]}=1\end{array}$$

According to the mathematical study above, the formula of the outbreak factor created by the SEIAPUFR model at time t in each period i is defined by

17 $$\:\left(B\right(t){)}_{i}=\begin{array}{l}\left|\frac{{\beta\:}_{i}S\left(t\right)\left(E\left(t\right)+A\left(t\right)+P\left(t\right)+U\left(t\right)\right)}{N{\gamma\:}_{i}\left[-\frac{\left(1+\rho\:\right)}{{\gamma\:}_{i}}E\left(t\right)+I\left(t\right)+A\left(t\right)+P\left(t\right)+U\left(t\right)+F\left(t\right)-\frac{{\vartheta\:}_{g}{N}^{\text{*}}}{{\gamma\:}_{i}}\text{log}\left(\frac{k}{F\left(t\right)}\right)\right]}\right|\end{array}$$

We define $\:\:{B}_{i}=\stackrel{-}{\left(B\right(t){)}_{i}}$as the average value of the outbreak factor for the period i.

### The spatial dissemination of COVID-19 throughout several urban areas

Given a collection of n interconnected communities, where the population of S,…,R are denoted as Sk,…,Rk correspondingly, we could lead to the following Eq. 

18 $$\:\begin{array}{l}\left\{\begin{array}{l}\dot{{s}_{k}}=\eta\:-{\beta\:}_{ik}{s}_{k}\left({e}_{k}+{a}_{k}+{p}_{k}+{u}_{k}\right)-{\sum\:}_{j=1}^{4}{C}_{J}\lambda\:{s}_{k}-g\tau\:{s}_{k}N,\\\:\dot{{e}_{k}}={\beta\:}_{ik}{s}_{k}\left({e}_{k}+{a}_{k}+{p}_{k}+{u}_{k}\right)-\sigma\:{e}_{k},\\\:\dot{{i}_{k}}=\sigma\:\alpha\:{e}_{k}-{\gamma\:}_{i}{i}_{k},\\\:\dot{{a}_{k}}=\sigma\:\left(1-\alpha\:\right){e}_{k}-{\gamma\:}_{i}{a}_{k},\\\:\dot{{p}_{k}}=\mu\:{e}_{k}-{\gamma\:}_{i}{p}_{k},\\\:\dot{{u}_{k}}=\left(1-\mu\:\right){e}_{k}-{\gamma\:}_{i}{u}_{k},\\\:\dot{{f}_{k}}=\vartheta\:g{N}^{*}\text{log}\left(\frac{k}{{f}_{k}}\right)+\rho\:{e}_{k}-{\gamma\:}_{i}{f}_{k},\\\:\dot{{r}_{k}}={\gamma\:}_{i}\left({i}_{k}+{a}_{k}+{p}_{k}+{u}_{k}\right)\end{array}\begin{array}{l}\\\:\\\:\\\:\\\:\\\:\\\:\\\:\end{array}\right.\end{array}$$

$\:{\beta\:}_{ik}$ represents the rate at which individuals in community *k* get infected. This expression should encompass both local community contacts and those linked with mobility, as stated by

19 $$\:{\beta\:}_{ik}=\sum\:_{j=1}^{n}{D}_{0,kj}^{S}\frac{\left({\theta\:}_{j}^{i}\sum\:_{l=1}^{n}{D}_{0,kj}^{i}{i}_{l}+{\theta\:}_{j}^{a}\sum\:_{l=1}^{n}{D}_{0,kj}^{a}{a}_{l}+{\theta\:}_{j}^{p}\sum\:_{l=1}^{n}{D}_{0,kj}^{p}{p}_{l}+{\theta\:}_{j}^{u}\sum\:_{l=1}^{n}{D}_{0,kj}^{u}{u}_{l}+{\theta\:}_{j}^{f}\sum\:_{l=1}^{n}{D}_{0,kj}^{f}{f}_{l}\right)}{\sum\:_{l=1}^{n}\left({D}_{0,lj}^{S}{s}_{l}+{D}_{0,lj}^{e}{e}_{l}+{D}_{0,lj}^{i}{i}_{l}+{D}_{0,lj}^{a}{a}_{l}+{D}_{0,lj}^{p}{p}_{l}+{D}_{0,lj}^{u}{u}_{l}+{D}_{0,lj}^{f}{f}_{l}+{D}_{0,lj}^{r}{r}_{l}\right)}$$

Where, the parameters $\:{\theta\:}_{j}^{Y}\left(Y\in\:\left\{i,a,p,u,f\right\}\right)$ represent the probability at which illness is transmitted from the five contagious groups, and these rates are reliant on the community, $\:{D}_{0,lj}^{Y}\left(Y\in\:\left\{s,e,i,a,p,u,f,r\right\}\right)$ represents the likelihood ( $\:\sum\:_{j=1}^{n}{D}_{0,lj}^{Y}=1$ for all k’s and Y’s) of individuals in epidemiology area Y, from community k, coming into interaction with individuals in community j, either as residents or while traveling from community l (note that j, k, and l might be the same). It should be noted that transmission has been presumed to vary depending on the frequency. On the other hand, the transmission rates $\:{\theta\:}_{j}^{Y}\left(Y\in\:\left\{i,a,p,u,f\right\}\right)\:$and monitoring parameters are considered to be potentially different for each community, which accounts for variations in disease transmission across different areas before containment measures were put in place($\:{\theta\:}_{j}^{Y})$.

### The fundamental reproductive number of the spatial mode

In close proximity to the DFE, the condition is characterized by a baseline population size (Nk) in community k, where all people are vulnerable to the illness. Additionally, all other epidemiological compartments are empty ($\:{e}_{k}={i}_{k}={a}_{k}={p}_{k}={u}_{k}={r}_{k}=0,\:and\:{f}_{k}={f}_{0}\:for\:all\:k{\prime\:}s$), the simplified systems $\:\dot{y}={J}_{0}y$ describes the dynamics of model (Eq. 18), where where $\:y={\left[{{s}_{k},e}_{k},{i}_{k},{a}_{k},{p}_{k},{u}_{k},{f}_{k},{r}_{k}\right]}^{T}$(where $\:k=1,\cdots\:,n$) and $\:{J}_{0}$ is the spatial Jacobian matrix.

20 $$\:{J}_{0}=\left(\begin{array}{l}\begin{array}{ccccccc}(\eta\:-\sum\:_{j=1}^{4}{C}_{j}\lambda\:-g\tau\:N)I&\:-{\beta\:}_{ik}sI&\:-{\psi\:}_{0}^{i}&\:-{\psi\:}_{0}^{a}&\:-{\psi\:}_{0}^{p}&\:-{\psi\:}_{0}^{u}&\:-{\psi\:}_{0}^{f}\:\:\:\:\:\:0\:\\\:0&\:{(\beta\:}_{ik}s-\sigma\:)I&\:{\psi\:}_{0}^{i}&\:{\psi\:}_{0}^{a}&\:{\psi\:}_{0}^{p}&\:{\psi\:}_{0}^{u}&\:{\psi\:}_{0}^{f}\:\:\:\:\:\:\:\:\:0\\\:0&\:\alpha\:\sigma\:I&\:-{\gamma\:}_{i}I&\:0&\:0&\:0&\:0\:\:\:\:\:\:\:\:\:\:\:0\\\:0&\:\alpha\:\left(1-\sigma\:\right)I&\:0&\:-{\gamma\:}_{i}I&\:0&\:0&\:0\:\:\:\:\:\:\:\:\:\:\:0\\\:0&\:\mu\:I&\:0&\:0&\:-{\gamma\:}_{i}I&\:0&\:0\:\:\:\:\:\:\:\:\:\:\:0\\\:0&\:\left(1-\mu\:\right)I&\:0&\:0&\:0&\:-{\gamma\:}_{i}I&\:0\:\:\:\:\:\:\:\:\:\:\:\:0\\\:0&\:\rho\:I&\:0&\:0&\:0&\:0&\:-\xi\:I\:\:\:\:\:\:\:\:0\end{array}\:\:\\\:\:\:\:\:\:\:\:\:\:\:\:\:\:\:\:\:\:\:\:\:0\:\:\:\:\:\:\:\:\:\:\:\:\:\:\:\:\:\:\:\:\:\:\:\:\:\:\:\:\:\:0\:\:\:\:\:\:\:\:\:\:\:\:\:\:\:\:{\gamma\:}_{i}I\:\:\:\:\:\:\:\:\:{\gamma\:}_{i}I\:\:\:\:\:\:\:{\gamma\:}_{i}I\:\:\:\:\:\:\:\:\:\:\:{\gamma\:}_{i}I\:\:\:\:\:\:\:0\:\:\:\:\:\:\:\:\:\:\:\:\:\:\:0\:\\\:\:\:\\\:\:\:\end{array}\right)$$

The identity matrix, denoted as I, and the null matrix, denoted as 0, both have a dimension of n. The matrices $\:{\psi\:}_{0}^{Y}\left(Y\in\:\left\{i,a,p,u,f\right\}\right)$ are provided by

21 $$\:{\psi\:}_{0}^{Y}=N{D}_{0}^{s}{\theta\:}^{Y}{\left({{\Gamma\:}}_{0}\right)}^{-1}{\left({D}_{0}^{Y}\right)}^{T}$$

here N is a diagonal matrix consisting of the population sizes N_k_ as its non-zero entries. The matrices $\:{D}_{0}^{Y}=\left[{D}_{0,lj}^{Y}\right]\left(Y\in\:\left\{s,i,a,p,u,f\right\}\right)$ reflect the contact terms that are regionally specifically and sub-stochastic, taking into account restrictions measures. The matrices $\:\:\:{\theta\:}^{Y}\left(Y\in\:\left\{i,a,p,u,f\right\}\right)$ are diagonal matrices that include the contact rates as their non-zero members $\:{\theta\:}_{j}^{Y}$, and $\:{{\Gamma\:}}_{0}$ is a diagonal matrix consisting of the components of vector $\:vN{D}_{0}^{s}$ as its non-zero entries, assuming that $\:v$ is a row vector of size *n* and is unitary.

Due to its block-triangular form, it is evident that $\:{J}_{0}$ has a single eigenvalue that is strictly negative, namely$\:-(-\eta\:+\sum\:_{j=1}^{4}{C}_{j}\lambda\:+g\tau\:N)$, with a multiplicity of *n*. Hence, the stability characteristics of the DFE of model (Eq. 18), which ascertain the possibility of sustained disease transmission in the presence of controls, are connected to the eigenvalues of a lower-dimensional spatial Jacobian connected to the infection subsystem, i.e., The subset of state factors that are directly associated with the transmission of the illness in this particular situation $\:\left\{{e}_{1},\cdots\:,{e}_{n},{i}_{1},\cdots\:,{i}_{n},{a}_{1},\cdots\:,{a}_{n},{p}_{1},\cdots\:,{p}_{n},{u}_{1},\cdots\:,{u}_{n},{f}_{1},\cdots\:,{f}_{n}\right\}$. It is important to know that the introduction of fading immunity does not alter the spectrum characteristics of the Jacobian matrix when assessed at the DFE. The Jacobian $\:{J}_{0}^{{\prime\:}}\:$is expressed as follows

22 $$\:{J}_{0}^{{\prime\:}}=\left(\begin{array}{l}\begin{array}{ccccccc}&\:-{\beta\:}_{ik}sI&\:-{\psi\:}_{0}^{i}&\:-{\psi\:}_{0}^{a}&\:-{\psi\:}_{0}^{p}&\:-{\psi\:}_{0}^{u}&\:-{\psi\:}_{0}^{f}\:\:\:\:\:\:\:\\\:&\:{(\beta\:}_{ik}s-\sigma\:)I&\:{\psi\:}_{0}^{i}&\:{\psi\:}_{0}^{a}&\:{\psi\:}_{0}^{p}&\:{\psi\:}_{0}^{u}&\:{\psi\:}_{0}^{f}\:\:\:\:\:\:\:\:\:\\\:&\:\alpha\:\sigma\:I&\:-{\gamma\:}_{i}I&\:0&\:0&\:0&\:0\:\:\:\:\:\:\:\:\:\:\:\\\:&\:\alpha\:\left(1-\sigma\:\right)I&\:0&\:-{\gamma\:}_{i}I&\:0&\:0&\:0\:\:\:\:\:\:\:\:\:\:\:\\\:&\:\mu\:I&\:0&\:0&\:-{\gamma\:}_{i}I&\:0&\:0\:\:\:\:\:\:\:\:\:\:\:\\\:&\:\left(1-\mu\:\right)I&\:0&\:0&\:0&\:-{\gamma\:}_{i}I&\:0\:\:\:\:\:\:\:\:\:\:\:\:\\\:&\:\rho\:I&\:0&\:0&\:0&\:0&\:-\xi\:I\:\:\:\:\:\:\:\:\end{array}\:\:\\\:\:\:\:\:\:\:\:\:\:\:\:\:\:\:\:\:\:\:\:\:\:\:\:\:\:\:\:\:\:\:\:\:\:\:\:\:\:\:\:\:\:\:\:\:\:\\\:\:\:\\\:\:\:\end{array}\right)$$

The asymptotic stability qualities of the DFE may be evaluated using a NGM technique. The spectral radius of the NGM offers the projection of the control reproduction number, $\:{R}_{0}$, which represents the average number of secondary infections caused by one contaminated people in a fully susceptible population with disease-containment measures in place. If the $\:{R}_{0}\:$is greater than 1, it indicates that the disease has the ability to establish itself in the community over time, resulting in endemic propagation. To calculate the value of $\:{R}_{0}$ for the model described by Eq. (18), it is possible to break down the Jacobian of the infection subsystem into a matrix that represents spatial transmission.

23 $$\:{V}_{0}=\left(\begin{array}{l}\begin{array}{ccccccc}&\:&\:&\:&\:&\:&\:\:\:\:\:\:\:\:\\\:&\:0&\:{\psi\:}_{0}^{i}&\:{\psi\:}_{0}^{a}&\:{\psi\:}_{0}^{p}&\:{\psi\:}_{0}^{u}&\:{\psi\:}_{0}^{f}\:\:\:\:\:\:\:\:\:\\\:&\:0&\:0&\:0&\:0&\:0&\:0\:\:\:\:\:\:\:\:\:\:\:\\\:&\:0&\:0&\:0&\:0&\:0&\:0\:\:\:\:\:\:\:\:\:\:\:\\\:&\:0&\:0&\:0&\:0&\:0&\:0\:\:\:\:\:\:\:\:\:\:\:\\\:&\:0&\:0&\:0&\:0&\:0&\:0\:\:\:\:\:\:\:\:\:\:\:\:\\\:&\:0&\:0&\:0&\:0&\:0&\:0\:\:\:\:\:\:\:\:\:\:\:\end{array}\:\:\\\:\:\:\:\:\:\:\:\:\:\:\:\:\:\:\:\:\:\:\:\:\:\:\:\:\:\:\:\:\:\:\:\:\:\:\:\:\:\:\:\:\:\:\:\:\:\\\:\:\:\\\:\:\:\end{array}\right)$$

and a transition matrix

24 $$\:{w}_{0}=\left(\begin{array}{l}\begin{array}{ccccccc}&\:&\:&\:&\:&\:&\:\:\:\:\:\:\:\\\:&\:{(\beta\:}_{ik}s-\sigma\:)I&\:0&\:0&\:0&\:0&\:0\:\:\:\:\:\:\:\:\:\:\\\:&\:\alpha\:\sigma\:I&\:-{\gamma\:}_{i}I&\:0&\:0&\:0&\:0\:\:\:\:\:\:\:\:\:\:\:\\\:&\:\alpha\:\left(1-\sigma\:\right)I&\:0&\:-{\gamma\:}_{i}I&\:0&\:0&\:0\:\:\:\:\:\:\:\:\:\:\:\\\:&\:\mu\:I&\:0&\:0&\:-{\gamma\:}_{i}I&\:0&\:0\:\:\:\:\:\:\:\:\:\:\:\\\:&\:\left(1-\mu\:\right)I&\:0&\:0&\:0&\:-{\gamma\:}_{i}I&\:0\:\:\:\:\:\:\:\:\:\:\:\:\\\:&\:\rho\:I&\:0&\:0&\:0&\:0&\:-\xi\:I\:\:\:\:\:\:\:\:\end{array}\:\:\\\:\:\:\:\:\:\:\:\:\:\:\:\:\:\:\:\:\:\:\:\:\:\:\:\:\:\:\:\:\:\:\:\:\:\:\:\:\:\:\:\:\:\:\:\:\:\\\:\:\:\\\:\:\:\end{array}\right)$$

It should be noted that $\:{J}_{0}={V}_{0}+{W}_{0}$. The spatial NGM, which encompasses a vast domain $\:{Q}_{0}^{L}$ and incorporates variables beyond the states-at-infection (namely, the exposed individuals $\:{e}_{i}$), may be expressed as follows:

25 $$\:\:{Q}_{0}^{L}=-{V}_{0}{\left({W}_{0}\right)}^{-1}=\left(\begin{array}{l}\begin{array}{ccccccc}&\:&\:&\:&\:&\:&\:\:\:\:\:\:\:\\\:&\:\:{Q}_{0}^{1}\:&\:\:{Q}_{0}^{2}\:&\:\:{Q}_{0}^{3}\:&\:\:{Q}_{0}^{4}\:&\:\:{Q}_{0}^{5}\:&\:\:{Q}_{0}^{6}\:\:\:\:\:\:\:\:\:\:\:\\\:&\:0&\:0&\:0&\:0&\:0&\:0\:\:\:\:\:\:\:\:\:\:\:\\\:&\:0&\:0&\:0&\:0&\:0&\:0\:\:\:\:\:\:\:\:\:\:\:\\\:&\:0&\:0&\:0&\:0&\:0&\:0\:\:\:\:\:\:\:\:\:\:\:\\\:&\:0&\:0&\:0&\:0&\:0&\:0\:\:\:\:\:\:\:\:\:\:\:\:\\\:&\:0&\:0&\:0&\:0&\:0&\:\:\:\:0\:\:\:\:\:\:\:\:\:\:\:\:\:\:\end{array}\:\:\\\:\:\:\:\:\:\:\:\:\:\:\:\:\:\:\:\:\:\:\:\:\:\:\:\:\:\:\:\:\:\:\:\:\:\:\:\:\:\:\:\:\:\:\:\:\:\\\:\:\:\\\:\:\:\end{array}\right)$$

Due to the distinctive block-triangular configuration of $\:{Q}_{0}^{L}$, the spatial NGM with a limited domain ($\:{Q}_{0}$, considering just $\:{e}_{i}$)) may be represented as $\:{Q}_{0}^{1}$. The control reproduction number may be determined by calculating the spectral radius of the NGM (with either a big or small domain) i.e.,

26 $$\:{R}_{0}=\rho\:\left(\:{Q}_{0}^{L}\right)=\rho\:\left({Q}_{0}\right)=\rho\:\left(\:{M}_{0}^{i}+{M}_{0}^{a}+{M}_{0}^{p}+{M}_{0}^{u}+{M}_{0}^{f}\right)\:\:\:$$

here, $\:{M}_{0}^{i},\:\:{M}_{0}^{a},\:{M}_{0}^{p},\:\:{M}_{0}^{u},\:\:{M}_{0}^{f}$, represent five matrices that describe the specific contributions of different factors (infected, diagnosed, respiration specific, non-respiration specific, and supervised sampling) to the next generation of infections in a neighborhood of the DFE when disease containment measures are in place.

### Fit of the model for the SARS-CoV-2 outbreak

Based on the varying population sizes, we deduce the model parameters. The estimated parameter values are based on various population sizes for the number of currently infected individuals with different SOI (asymptomatic or paucisymptomatic, quarantined at home, roughly corresponding to variables I(t) and A(t) in our model; symptomatic and respiratory specific, roughly corresponding to variable P (t) in our model; symptomatic and non-respiratory specific, roughly corresponding to variable U (t) in our model, the number of supervised sampling in infected and vaccinated population in the specific period, roughly corresponding to variable F (t) in our model), and the number of diagnosed individuals with non-imperfect test accuracy who recovered (approximately corresponding to the quantity $\:{\int\:}_{0}^{t}{\gamma\:}_{i}\left(I\left(\psi\:\right)+A\left(\psi\:\right)+P\left(\psi\:\right)+U\left(\psi\:\right)\right)d\psi\:$ that can be computed based on our model). We fit the model to the number of presently infected cases, respiratory cases, and supervision sampling.

We use a best-fit technique to determine the settings that minimize the sum of the squares of the errors locally. The model has a massive number of state variables and a large number of unknown parameters whose numerical calculation is a difficult task; it is probable that an unlimited number of distinct parameter sets might be produced that all match the data equally well. On the other hand, our parameters are control tuning knobs whose settings should accurately recreate the data. The model parameters were fitted using repeated local minimization of the sum of the squares of the errors, based on a priori epidemiological and clinical knowledge of relative parameter magnitude (as stated above) and beginning from a random initial guess. The parameters were updated during the simulation based on the sequential steps taken by policymakers, each of increasing strength.

## Results

### Parameter estimation methods

The parameter estimation (PE) methodology is a beneficial and newly popular method for determining the nearest parameter values to the actual data while producing the curve that best matches the real data. The PE approach calculates the most realistic parameter values for the established mathematical model. The numerical values of the parameters in the majority of the studies in the literature are the estimated parameter values utilized in the preceding articles^36–42^). This strategy improves the utility of our research by calculating model-specific parameter values. The PE methodology is a highly effective way to find the best appropriate curve for the actual data while delivering parameter values as near to the true values as possible.

**Fig. 2:**
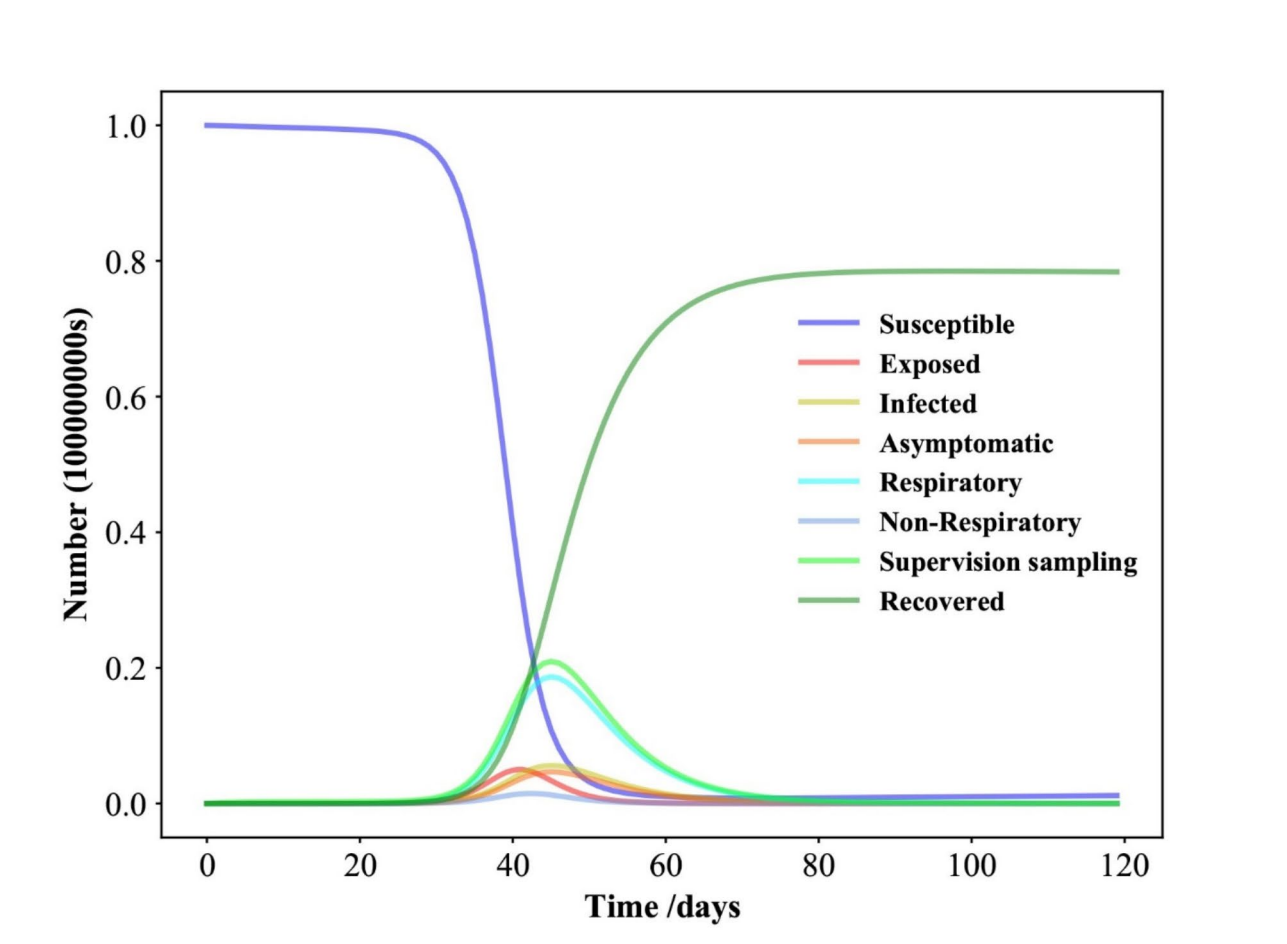
Time evolution for each population in Shenzhen in the SEIAPUFR model for *N* = 17,560,000. Each generation chooses all the sites randomly and according to their state.

### Numerical simulation of SEIAPUFR model of COVID-19 in each city

The product of the number of infected and susceptible persons determines the pace of the rise in the number of infections. Understanding the SEIAPUFR model system reveals the alarming rise in infection rates worldwide. As a result of sick persons moving throughout the world, the number of infected people has increased, resulting in a rise in the vulnerable population^43^). This creates a positive feedback loop, causing the number of actively infected patients to increase rapidly. As a result, during a surge period, the number of vulnerable persons increases, as does the number of infected individuals.

### Numerical simulation results of the SEIAPUFR model of COVID-19 in Shenzhen

The proportion of the population at each stage at any given time is determined as follows: *N* = 17,560,000, N^∗^ = 1384, E(0) = 1, A(0) = 1, P (0) = 0, U (0) = 1, I(0) = 1384, F (0) = 1, R(0) = 1369, S = N − I − R − E − A − F − U, C0 = 0, C1 = 0.95, C2 = 0.95, C3 = 0.95, η = 0.001, λ = 0.96, σ = 0.4, α = 0.6, ε = 1- µ, β = 0.25, γ = 0.3, µ = 0.8, ρ = 0.9, and k = 469,917,τ = 0.7,g = 23, which were reported by^44^).

We tested the model mentioned above with *N* = 17,560,000 and found the following time evolution for each population of the SEIAPUFR model (Fig. 2). Each population’s size is determined by the density of infected persons every day. When we look at the time evolution in Fig. 2, we can see that the differential equation models have a similar form as in^45^). The susceptible population decreases as the recovery population grows over time until the disease spread stops and a stationary value is reached. The exposed, infected, asymptomatic, respiratory-specific model, non-respiratory specific model, and guided sampling in infected populations grow until they reach a maximum value, then fall until they reach a null value at reducing epidemics. In this approach, our model may be used to replicate the SEIR model’s behavior. To better understand the policy resolution of these fundamental framework cases, we run various tests with different values of N.

**Fig. 3:**
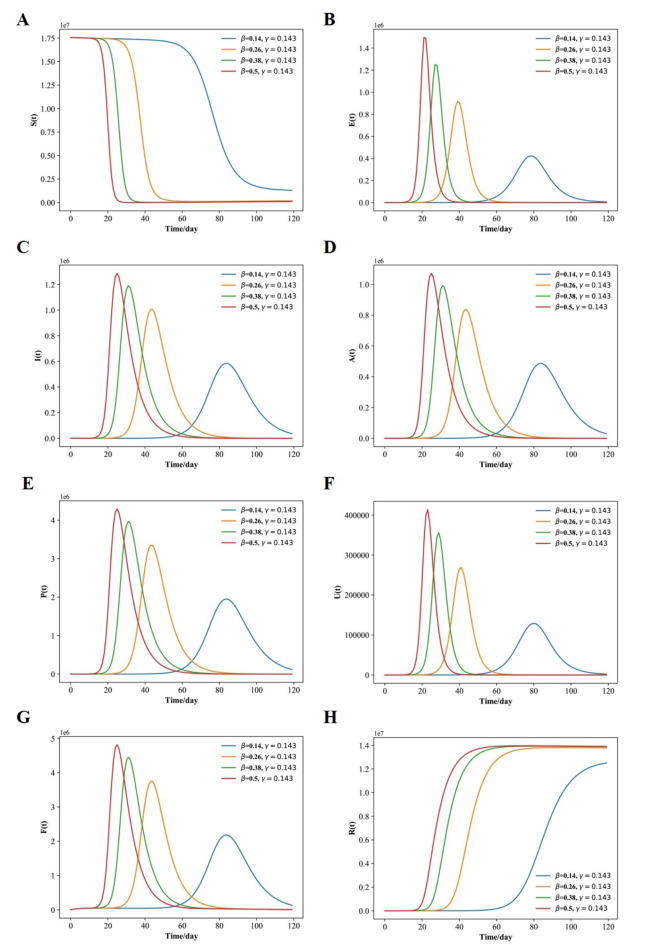
Fitted and predicted epidemic evolution. (**A**–**H**), Population dynamics of susceptible; exposed; infected; asymptomatic, respiratory specific model; non-respiratory specific model; supervised sampling in infected individuals, and healed class of the considered model using various values of β and γ.

The above settings were used to replicate the model and produce the graphs shown in Fig. 3(A-H). A comparison of the curves produced by the SEIAPUFR model is shown. The current number of infected (including all phases), recovered, and diagnostic cases are all accurately replicated. The difference between the two curves can be explained as follows:

The model takes into account infections of various severity levels (for example, P (t) is the number of respiratory diseases that must be considered when examining the pandemic’s flatten curve, and F (t) is the number of surveillance samples that must be considered when estimating the number of infectious individuals on a given day). In contrast, Fig. 2 depicts the epidemic progression predicted by the model for the COVID-19 outbreak, after day 34. In the presence of perfect test accuracy, countermeasures, and vaccine effectiveness (such as lockdowns, social distancing, hygiene recommendations, and the use of face masks), we have β ∈ {0.14, 0.2, 0.25, 0.5} and γ = 0.3, and the model predicts an evolution that leads to 220, 000; 500, 000; 700, 000; 1, 500, 000; of the individuals having been exposed by the virus, and 200, 000; 389,000; 500, 000; 810, 000 of the individuals having Asymptomatic, 600,000; 1, 200, 000; 1, 600,000; 2, 700, 000 of the population having respiratory specific because of the indoor and outdoor airborne infections,150,000; 310,000; 420,000; 680,000 of the population having non-respiratory specific, 600,000; 1,400,000; 1,800,000; 3,100,000 of the population having supervision sampling across a 120-day horizon (Fig. 3A-H) for β ∈ {0.14, 0.2, 0.25, 0.5}.

**Fig. 4:**
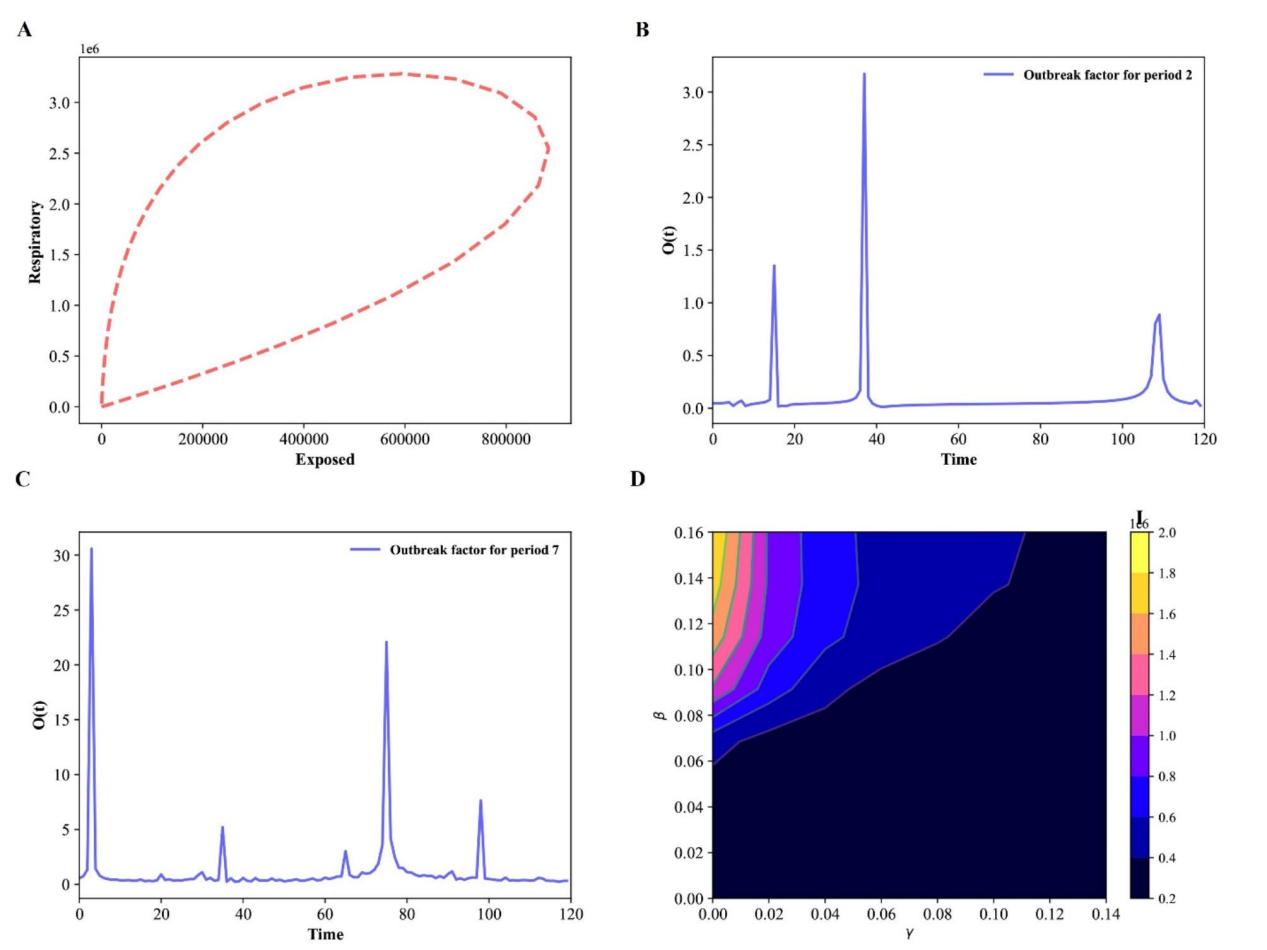
Epidemic evolution predicted by the model for the COVID-19 outbreak, when *N* = 17,560,000. (**A**) The relationship between respiratory population and exposed population during the evolution control of the pandemic; (**B**,**C**) Shown an outbreak component, in periods two and three; (**D**) Time development of the density of the infected population for various values of the parameters β and γ.

**Fig. 5:**
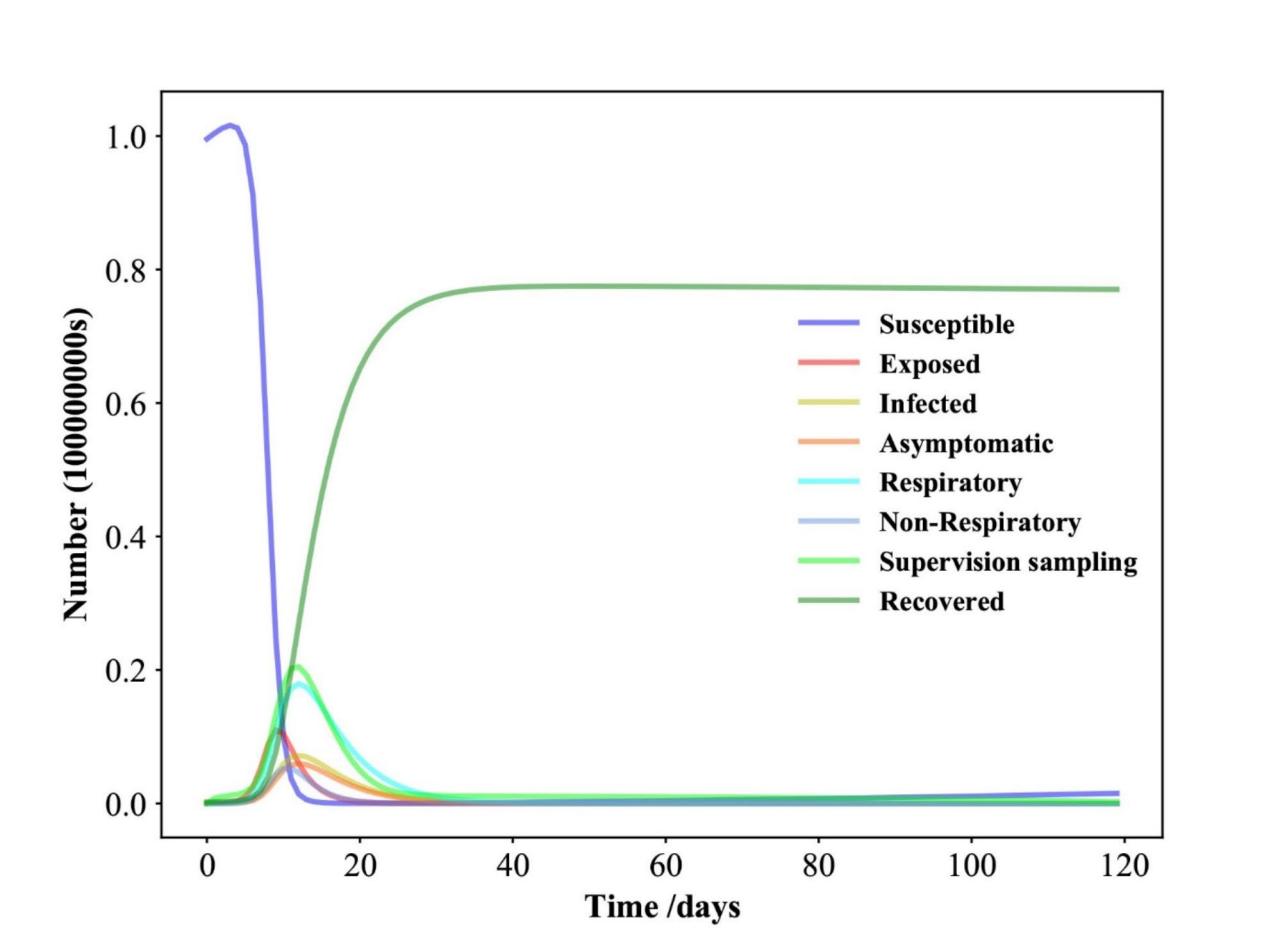
Time evolution for each population in Shanghai in the SEIAPUFR model for *N* = 24,870,895. Each generation chooses all the sites randomly and according to their state.

and γ = 0.3, respectively. For β = 0.5, the peak of concurrently exposed, infected, asymptomatic, respiratory specific, non-respiratory specific, and supervision sampling individuals occurs around 18 days and amounts to 53.99% of the population; however, the peak of concurrently exposed, infected, asymptomatic, respiratory specific, non-respiratory specific, and supervision sampling individuals for β = 0.14 occurs later, around 62 days, and amounts to 10.93% of the population.

The difference between sub-populations of exposed individuals and respiratory-specific individuals evolves, as shown in Fig. 4A, and it’s interesting to note that respiratory-specific sub-populations increased with exposed sub-populations at different time and then started to decrease around (750000; 1600000), it means that many steps, such as the use of face masks, social distance, the deployment of an effective vaccine, different degrees of testing, and the building of herd immunity, are being taken from this point forward to flatten the transmission curve.

**Fig. 6:**
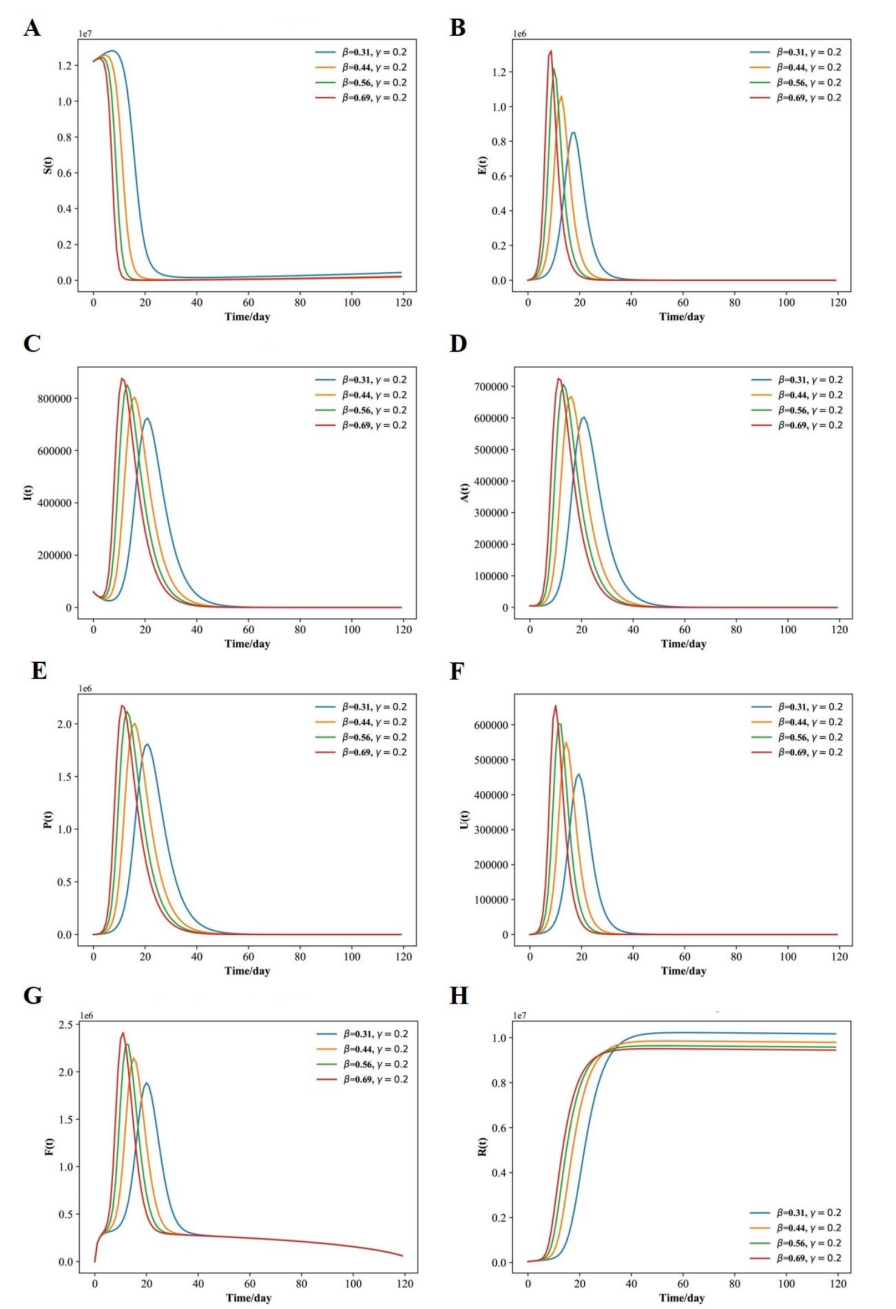
Fitted and predicted epidemic evolution. (**A**–**H**) Population dynamics of susceptible; exposed; infected; asymptomatic, respiratory specific model; non-respiratory specific model; supervised sampling in infected individuals and healed class of the considered model using various values of β, and γ, *N* = 24,870,895.

**Fig. 7:**
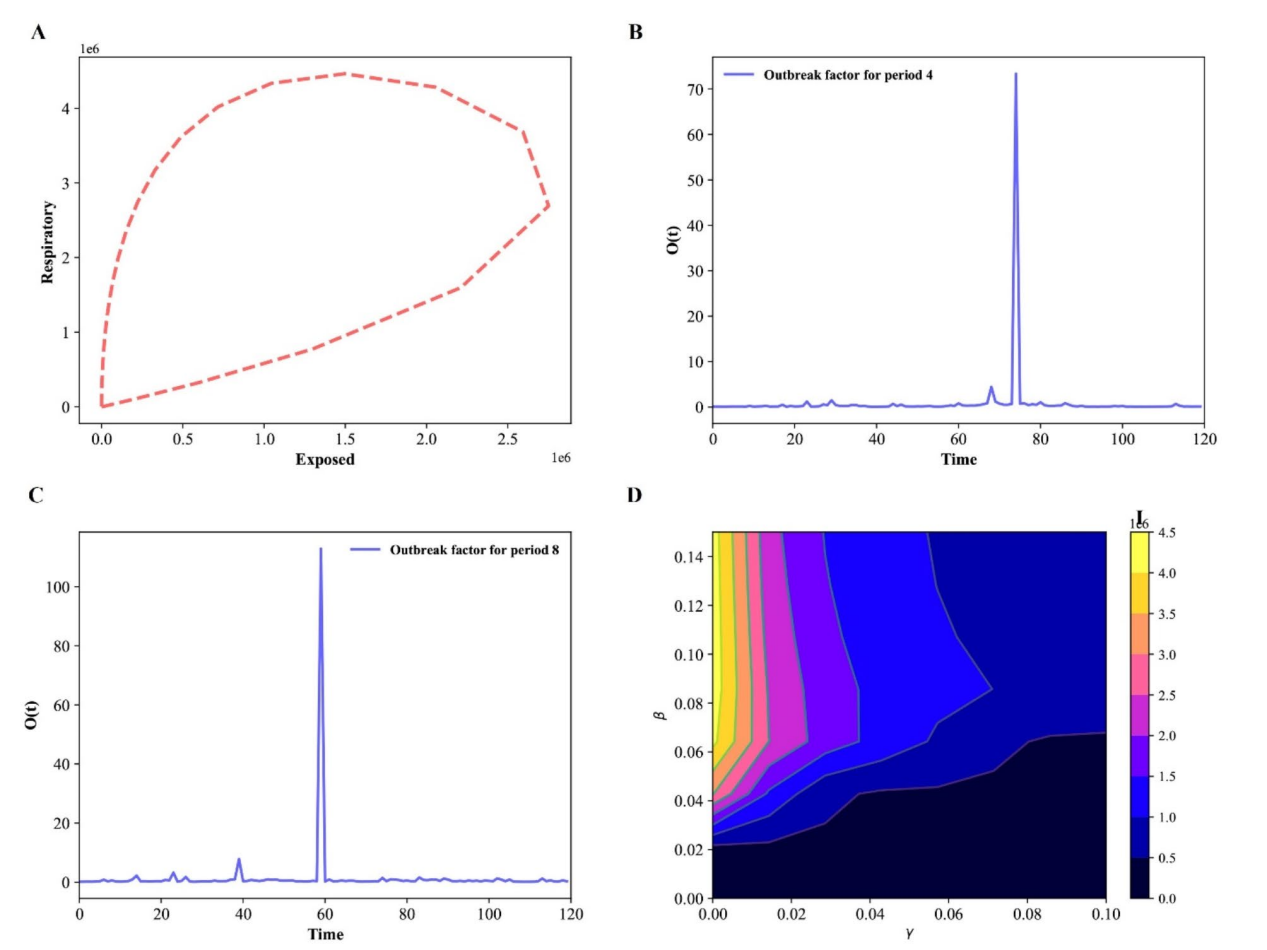
Epidemic evolution predicted by the model for the COVID-19 outbreak, when *N* = 24,870,895. (**A**) The relationship between the respiratory population and exposed population during the evolution control of the pandemic; (**B**,**C**) Shown an outbreak component in periods four and six; (**D**) Time development of the density of the infected population for various values of the parameters β and γ.

If respiratory sub-populations are managed early on, the transmission may be lowered over time. As a result of our model predictions, we can conclude that the respiratory-specific sub-population has a potential effect on flattening the curve during pandemic transmission, which would prevent the health care system from becoming overwhelmed because the peak number of beds occupied at any given time would be lower under a flatter curve, would slow the momentum of the outbreak, reducing the overshoot of cases after the outbreak peak, and would allow time to develop clinical care methods and capability, as well as to analyze treatments with the flawless test accuracy and improve vaccine efficacy^46,47^).

**Fig. 8:**
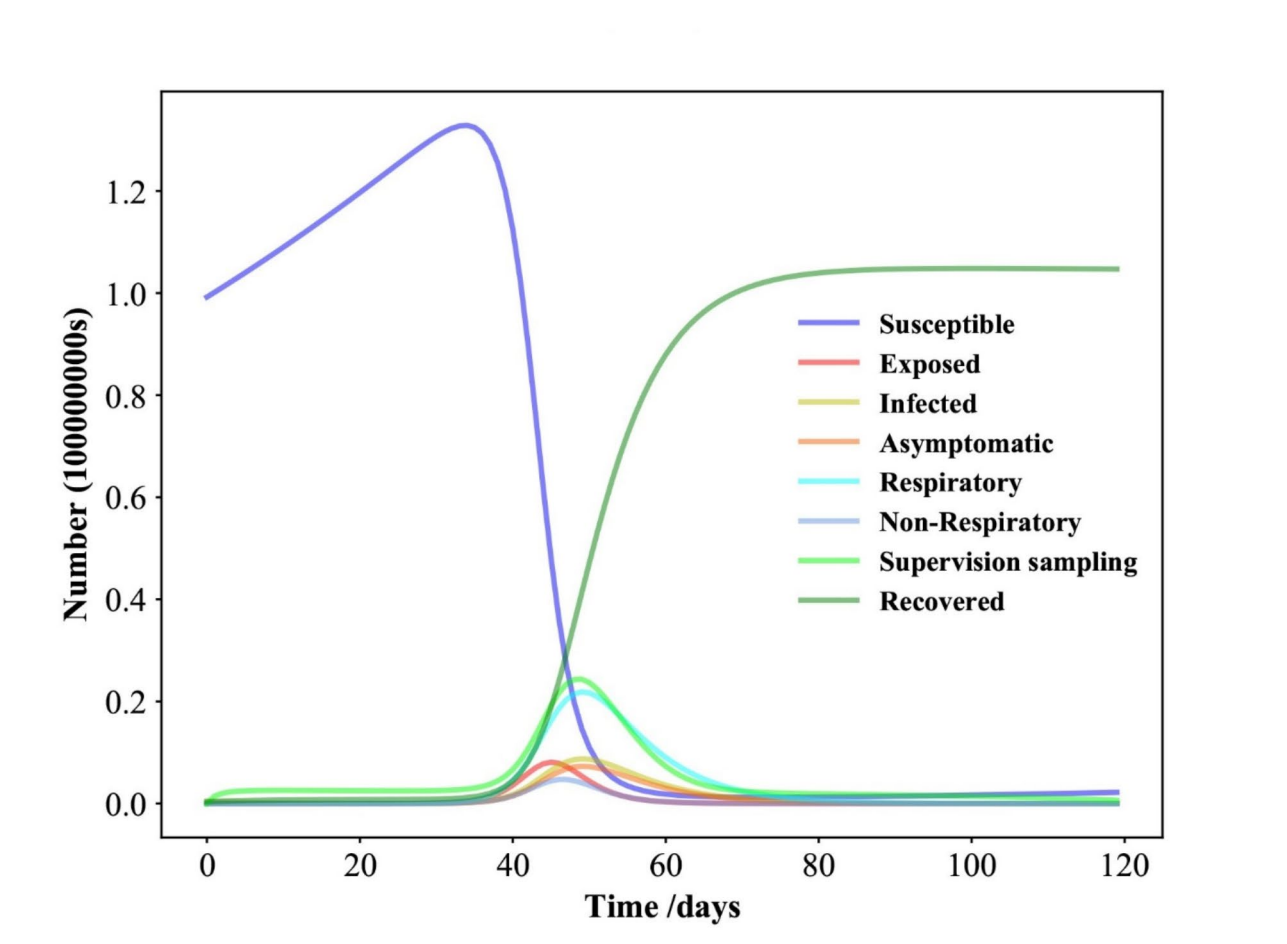
Time evolution for each population in Wuhan in the SEIAPUFR model for *N* = 12,326,518. Each generation chooses all the sites randomly and according to their state.

The numerical simulation of the outbreak factor in periods 2 and 3 is shown in Fig. 4B, C. Before the initial peak (41, 50 days) and after about 61 days, respiratory-specific control, countermeasure, and perfect test accuracy regulations can delay the development of the pandemic. In both cases(periods two and three), the higher epidemic values are focused around t = 60 days. Based on this observation, we may assume that with less control (t < 70), the infected curve has a larger peak and, after that, the spread cases for t = 80, showing that the density of infected people is higher, but the infection ends sooner, even when control measures are loosened. Furthermore, if no reasonable control measures are implemented, an exponential pandemic peak will inevitably occur over time. Based on the varied values of β and γ, we plot some curves of the number of infectious individuals during the pandemic transmission (Fig. 4D). When we apply a higher control (β < 0.03), we notice an expansion of the dark blue zone in Fig. 4D, which is between a blue region and a dark portion. This scenario exhibits various peaks separated by a portion of lower I value, describing a wave of infections^45^). Furthermore, the color scale in Fig. 4D reflects the amount of I.

**Fig. 9:**
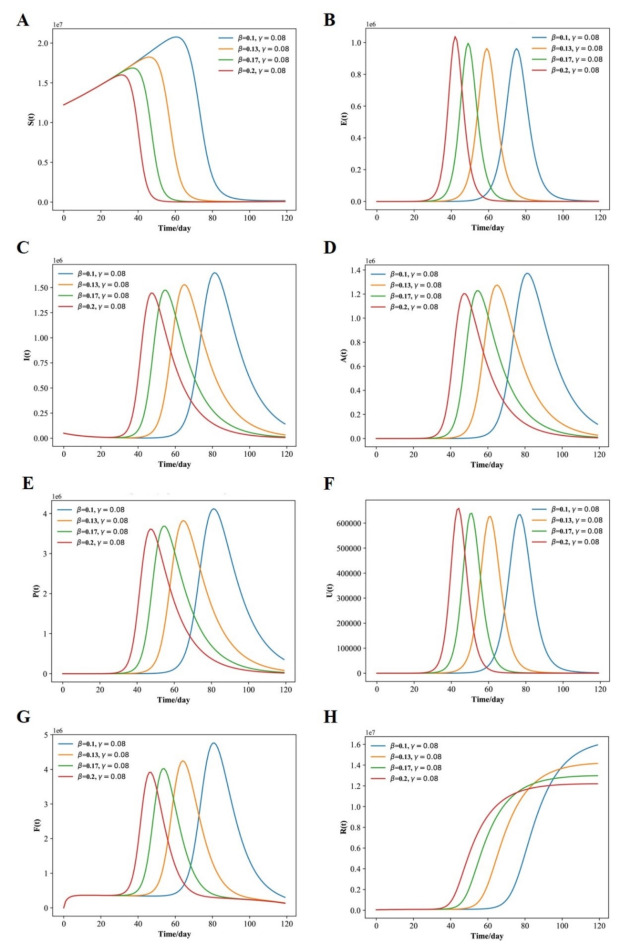
Fitted and predicted epidemic evolution. (**A**–**H**) Population dynamics of sus- ceptible; exposed; infected; asymptomatic, respiratory specific model; non-respiratory specific model; supervised sampling in infected individuals and healed class of the considered model using various values of β, and γ, *N* = 12,326,518.

**Fig. 10:**
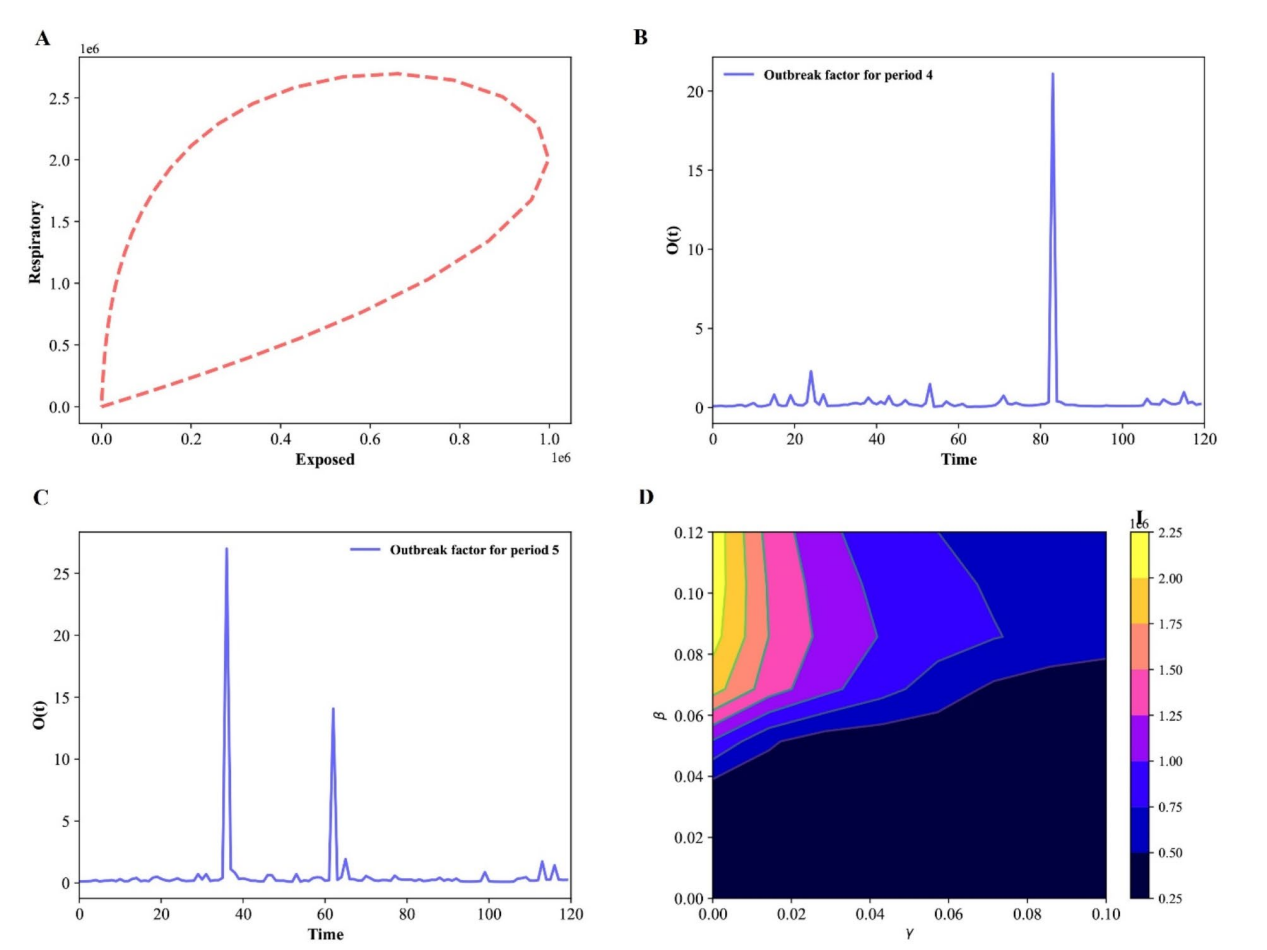
Epidemic evolution predicted by the model for the COVID-19 outbreak, when *N* = 12,326,518. (**A**) The relationship between the respiratory population and exposed population during the evolution control of the pandemic; (**B**,**C**) Shown an outbreak component in periods two and five; (**D**) Time development of the density of the infected population for various values of the parameters β and γ.

The colored zones represent combinations of β and γ for which the pandemic continues indefinitely. Meanwhile, we have scenarios like epidemic extinction in the yellow zone, despite the relaxation of control efforts and reinfection. Greater values of I correspond to higher values of β. As we can see, even at a greater γ = 0.14, the value of infection is I = 0.0. As the value of β rises, the value of disease I fall for all γ values.

**Fig. 11:**
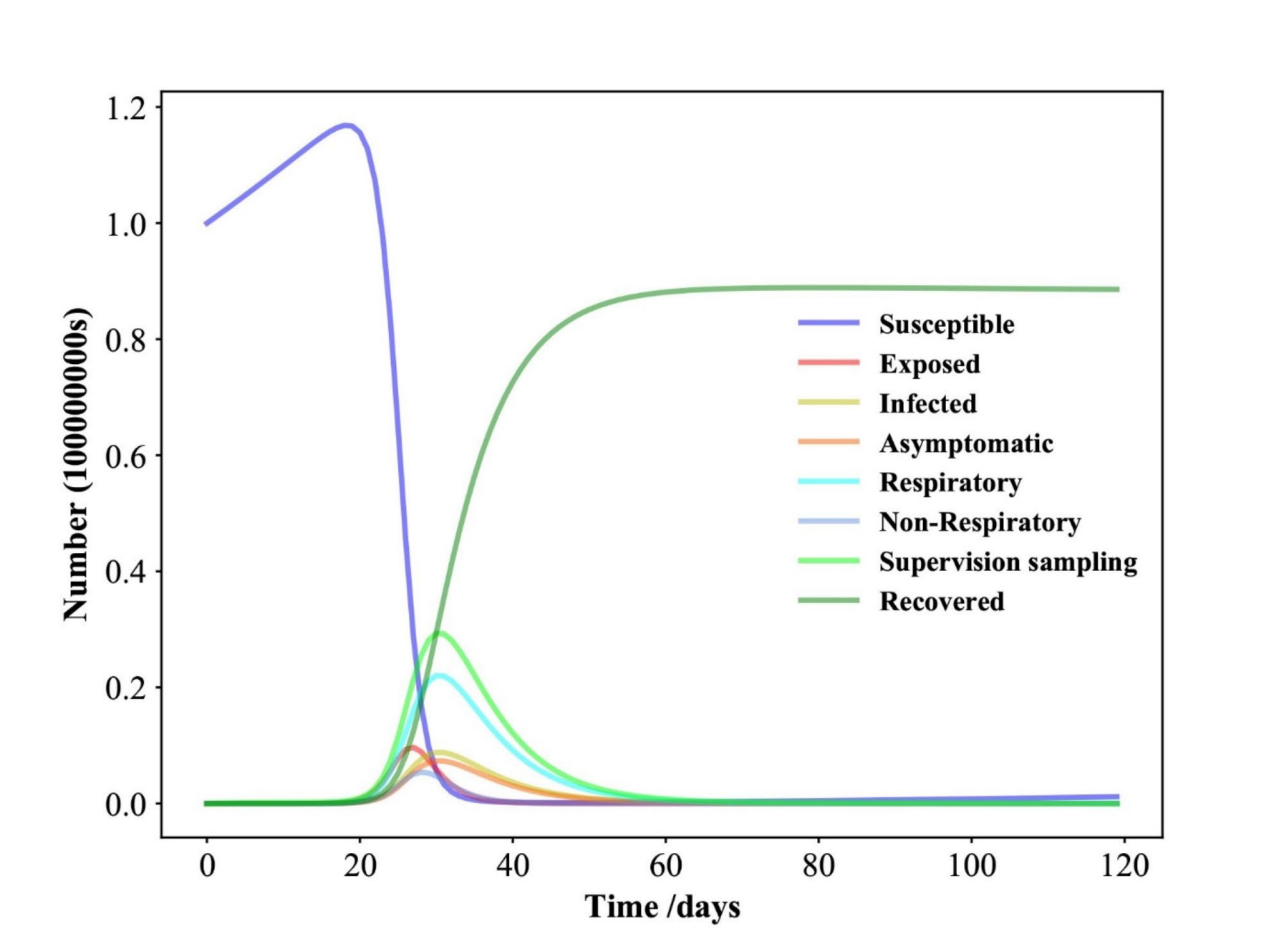
Time evolution for each population in Guangzhou in the SEIAPUFR model for *N* = 18,676,605. In each generation, all the sites are chosen randomly and according to their state.

The black region, for lower values of β and higher values of γ, can be explained by the fact that, with control measures (lower values of γ), a greater number of people are infected at the start of the spread, but when the people become susceptible again, there are no more infected people to perpetuate the disease spread in the exposed compartment. From the above finding, we can deduce that the reduction arises solely once the amount of susceptibles diminishes, which was solely due to the stringent measures implemented by the Chinese government in Shenzhen. China implemented quarantine measures for possible patients, regulated individuals’ mobility, and limited overseas travel. Social separation was extensively observed, and the majority of individuals wearing face masks. The actual infection rates have declined more significantly than anticipated by the SIR model. The result shown in Figs. 3, and 4 indicate that the Chinese government has effectively mitigated the effects of COVID-19’s propagation in Shenzhen.

### Numerical simulation results of the SEIAPUFR model of COVID-19 in Shanghai

The proportion of the population at each stage at any given time is determined as follows: *N* = 24,870,895, N ^∗^ = 59,342, E(0) = 1, A(0) = 5396, P (0) = 0, U (0) = 1, I(0) = 59,342, F (0) = 1, R(0) = 45,873, S = N − I − R − E − A − F − U, C0 = 0, C1 = 0.96, C2 = 0.96, C3 = 0.95, η = 0.001, λ = 0.97, σ = 0.4, α = 0.6, ε = 1- µ, β = 0.691, γ = 0.2, µ = 0.6, ρ = 0.8, and k = 469,917,τ = 0.7,g = 23, which were reported by^44^).

**Fig. 12:**
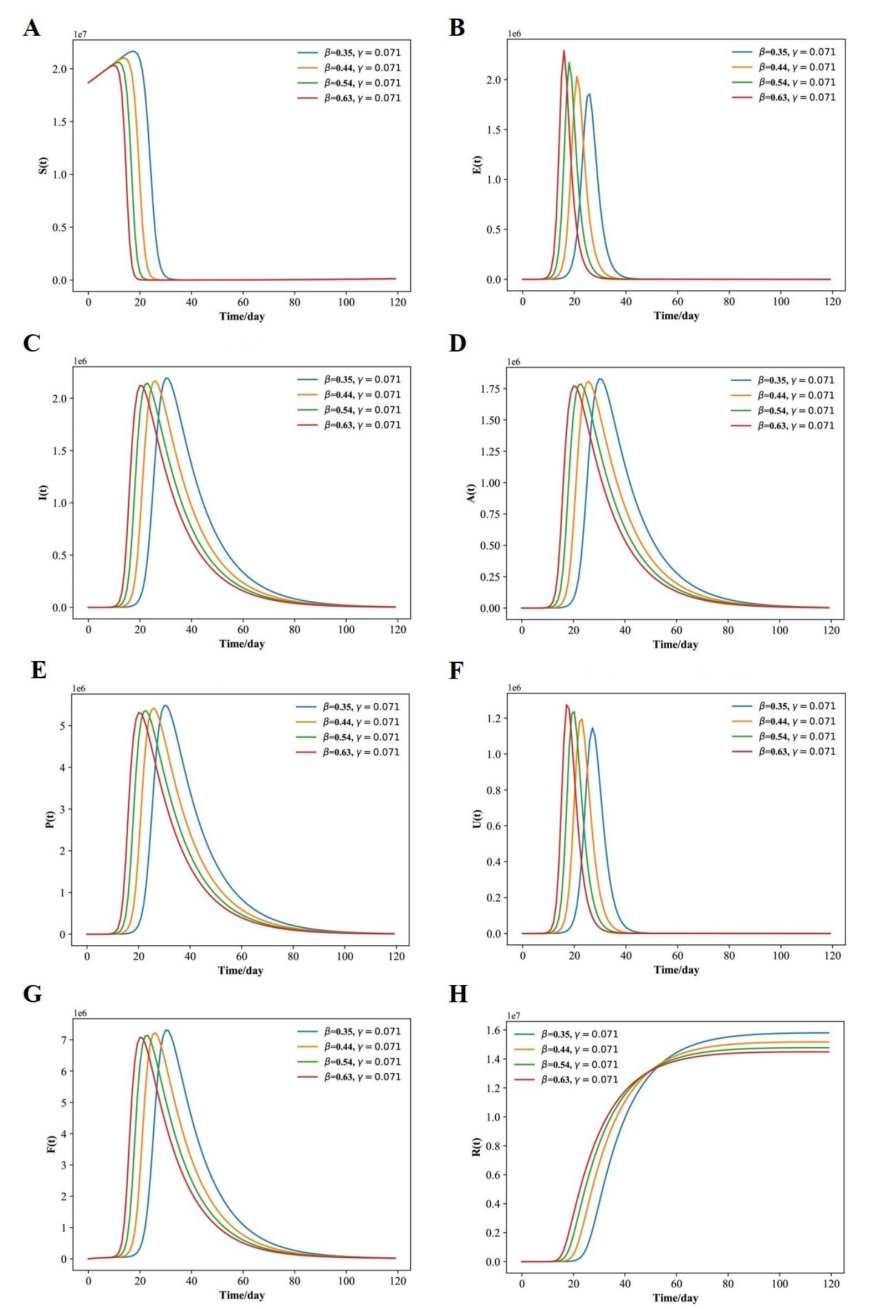
Fitted and predicted epidemic evolution. (**A**–**H**), Population dynamics of susceptible; exposed; infected; asymptomatic, respiratory specific model; non-respiratory specific model; supervised sampling in infected individuals and healed class of the considered model using various values of β, and γ, *N* = 18,676,605.

**Fig. 13:**
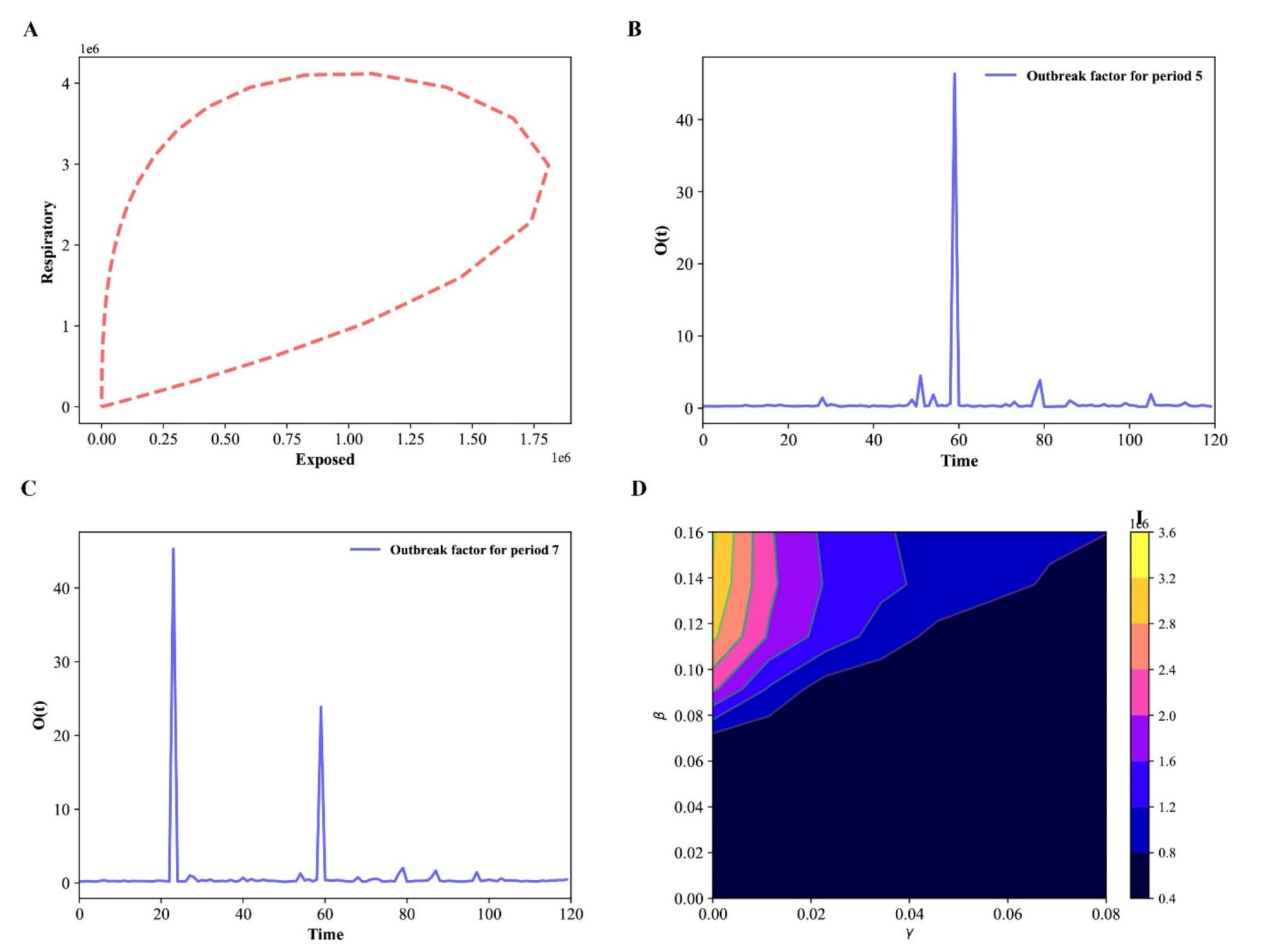
Epidemic evolution predicted by the model for the COVID-19 outbreak, when *N* = 18,676,605. (**A**) The relationship between respiratory population and exposed population during the evolution control of the pandemic; (**B**,**C**) Shown an outbreak component, in periods five and seven; (**D**) Time development of the density of the infected population for various values of the parameters β and γ.

From the above parameters, analyzing the time evolution presented in Fig. 5, we observe the similar results when *N* = 24,870,895. The susceptible population decreases as the recovery population increases over time until a stationary value achieved when the disease spread ends. The exposed, the infected, the asymptomatic, the respiratory specific model, non-respiratory specific model, and supervised sampling in infected populations increase until a maximum value and then they decrease and assume a null value in the end of the epidemics. Conversely, Fig. 5 shows the epidemic evolution that would have been predicted by the model for the COVID-19 outbreak, after 25 days.

The Fig. 6A-H shows that, with use of face marks, social-distancing countermeasures, vaccine effectiveness, and perfect test accuracy after days 5, 10, 15, 20 having a mild effect, β ∈ {0.31, 0.44, 0.56, 0.69} and γ = 0.2. Hence, the peak is delayed for each compartment and reduced in amplitude, because of vaccine effectiveness and perfect test accuracy. Over a 120-day horizon, as shown in Fig. 6C, the model predicts an evolution that leads to a peak in the number of concurrently infected individuals around days 15, 17, 19, 21, amounting to 6.07%, 6.47%, 6.79%, 7.07% of the population for β ∈ {0.31, 0.44, 0.56, 0.69} and γ = 0.2, respectively. Eventually, 15.68%, 16.88%, 18.09%, 19.29% of the population have respiratory specific with the virus because of the contagion and 10.45%, 10.85%, 11.25%, 12.06% of the population have respiratory specific for β ∈ {0.31, 0.44, 0.56, 0.69} and γ = 0.2, respectively (Fig. 6F). The fractions of patients in supervision sampling, are shown in Fig. 6G, and reach their peak on days 5, 10, 15, 20, amounting to 17.29%, 18.5%, 19.7%, 20.9% of the population for β ∈ {0.31, 0.44, 0.56, 0.69} and γ = 0.2. The adopted use of face marks, social-distancing countermeasures, vaccine effectiveness, and perfect test accuracy, although mild, have some impact and help gain more time to strengthen and supply the health care system, but is still insufficient.

The Fig. 7A shows how the different between sub-populations of exposed individuals and respiratory specific individuals evolve over time, and it is interesting to notice that the respiratory specific sub-populations increased with exposed sub-populations at a different time and started to decrease around a point (2800000; 3000000),it implies that several measures are being made from this point on to flatten the transmission curve, such as the use of face masks, social distance, the deployment of an effective vaccine, varying degrees of testing, and the establishment of herd immunity. In particular, if the respiratory sub-populations are early controlled the transmission could be reduced over the time. The Fig. 7B, C shows the numerical simulation of outbreak factor in periods 4 and 6. In periods 4 and 6, the respiratory specific control, countermeasure, and perfect test accuracy rules can slow the spread of pandemic before the first peak(39 day), and latter around 54, 59, 81 days. In both cases(periods 4 and 6), the highest values of the epidemic are concentrated around t = 38 days. Based on this observation, we may assume that with less control (t < 40), the infected curve has a larger peak and, after it, the spread cases for t = 50, 59, 81, showing that the density of infected people is higher but the infection ends sooner, even when control measures are loosened. Furthermore, if no roughly control measures are put in place, we will constantly observe an exponential pandemic peak over time. The Fig. 7D show the number of infectious individuals during the transmission of the pandemic based on the different values of β, and γ. When apply a lower control (β = 0.2), we notice an enlargement of the blue zone in Fig. 7D. This scenario exhibits various peaks separated by a portion of lower I values, which describes a scenario of a wave of infections^45^). Furthermore, the color scale in Fig. 7D reflects the amount of I. The colored zones represent combinations of β and γ for which the pandemic continues indefinitely.

Nevertheless, in the orange zone, we are seeing events connected to the extinction of the epidemic, despite the relaxation of control efforts and reinfection. Greater values of I correspond to higher values of β. As the value of β rises, the value of infection I falls for all γ values. The black region, for lower values of β and higher values of γ, can be explained by the fact that a greater number of people are infected at the start of the spread, but when the people become susceptible again, there aren’t enough infected people to keep the disease spreading in the exposed compartment. The steps implemented were effective, as evidenced by the decrease in the actual amount of cases aligning with the model projections. Therefore, the actions currently implemented are essential to control the outbreak and may not be relaxed. Instead, they ought to be increasingly stringent. The effectiveness of the mandated vaccine may be diminished by extensive testing and contact tracing, which would significantly aid in the swift end of this pandemic.

### Numerical simulation results of the SEIAPUFR model of COVID-19 in Wuhan

The proportion of the population at each stage at any given time is determined as follows: *N* = 12,326,518, N ^∗^ = 50,423, E(0) = 1, A(0) = 0, P (0) = 1, U (0) = 0, I(0) = 50,423, F (0) = 1, R(0) = 46,551, S = N − I − R − E − A − F − U, C0 = 0, C1 = 0.96, C2 = 0.96, C3 = 0.95, η = 0.001, λ = 0.97, σ = 0.4, α = 0.6, ε = 1- µ, β = 0.2, γ = 1/7, µ = 0.6, ρ = 0.8, and k = 469,917,τ = 0.7,g = 23, which were reported by^44^).

From the above parameters, analyzing the time evolution presented in Fig. 8, we observe the similar results when *N* = 12,326,518. The susceptible population decreases as the recovery population increases over time until a stationary value achieved when the disease spread ends. The exposed, the infected, the asymptomatic, the respiratory specific model, non-respiratory specific model, and supervised sampling in infected populations increase until a maximum value and then they decrease and assume a null value in the end of the epidemics. Conversely, Fig. 8 shows the epidemic evolution that would have been predicted by the model for the COVID-19 outbreak, after 41 days. If there is a minor increase in the future population of vulnerable population no significant effects will ensue. Nonetheless, a significant epidemic of the virus can yield devastating consequences.

The Fig. 9A-H shows that, with stronger use of face masks with vaccine and perfect test accuracy, able to yield β ∈ {0.1, 0.13, 0.17, 0.2} and γ = 0.08, the peak is delayed, because the increase in the number of new infected is reduced so much that it soon becomes a decrease. Over a 120-day horizon, as shown in Fig. 9C, the model predicts an evolution of the situation that leads to a peak in the number of concurrently infected individuals around day 75, 60, 50, 40, amounting to 20.28%, 18.65%, 17.84%, 16.22% of the population for β ∈ {0.1, 0.13, 0.17, 0.2} and γ = 0.08, respectively. Eventually, 52.73%, 47.05%, 45.43%, 44.62% of the population have respiratory specific with the virus and 34.07%, 31.64%, 30.83%, 29.21% of the population have non respiratory specific for β ∈ {0.1, 0.13, 0.17, 0.2} and γ = 0.08 because of the non control of respiratory specific individuals. The fraction of patients in need of supervision sampling, as shown in Fig. 9G, reach their peaks on day 75, 60, 50, 40, amounting to 58.41%, 55.17%, 52.73%, 51.92% of the population.

The Fig. 10A shows how the different between sub-populations of exposed individuals and respiratory specific individuals evolve over time, and it is interesting to notice that the respiratory specific sub-populations increased with exposed sub-populations at a different time and started to decrease around a point (1100, 000; 2100, 000), it suggests that numerous efforts are being taken to flatten the transmission curve from this point on, such as the use of face masks, social distance, the deployment of an effective vaccine, various degrees of testing, and the development of herd immunity. In particular, if the respiratory sub- populations are early controlled the transmission could be reduced over the time. The Fig. 10B, C shows the numerical simulation of outbreak factor in periods 2 and 5. In periods 2 and 5, the respiratory specific control, countermeasure, and perfect test accuracy rules can slow the spread of pandemic before the first peak(78 day), and latter around 98 days. In both cases(periods 2 and 5), the higher outbreak values are clustered around t = 78, 98 days. Based on this observation, we may conclude that with less control (70 < t < 100), the infected curve has a bigger peak and, after it, the spread cases for t = 78, 98, showing that the density of infected people is higher but the infection ends sooner, even when control measures are loosened. Furthermore, we will always witness an exponential epidemic peak over time if no reasonable control measures are put in place. The Fig. 10D show the number of infectious individuals during the transmission of the pandemic based on the different values of β, and γ. When a greater control is given, we witness an expansion of the dark blue zone (γ > 0.1) in Fig. 10D, which is between a blue region and a dark section. Following the color scale, this scenario exhibits various peaks separated by a portion of lower I values, which indicates a scenario of a wave of infections^45^). Furthermore, in Fig. 10D, the color scale represents the amount of I. The colored zones represent combinations of β and γ for which the pandemic does not terminate over time. Moreover, in the orange zone, we are seeing scenarios connected to the extinction of the epidemic, despite the relaxation of control efforts and the reinfection. Greater values of I are present for higher levels of β. As we can see, even at larger γ > 0.1 values, the value of infection I = 0.0. As the value of β grows, the value of infection I falls for all values of γ. The black region, for lower values of β and higher values of γ, can be explained by the fact that, with lower control measures (lower values of γ), a greater number of individuals are infected at the start of the spread, and then, when the individuals become susceptible again, there will be no more infected individuals to perpetuate the disease spread in the exposed compartment.

**Fig. 14:**
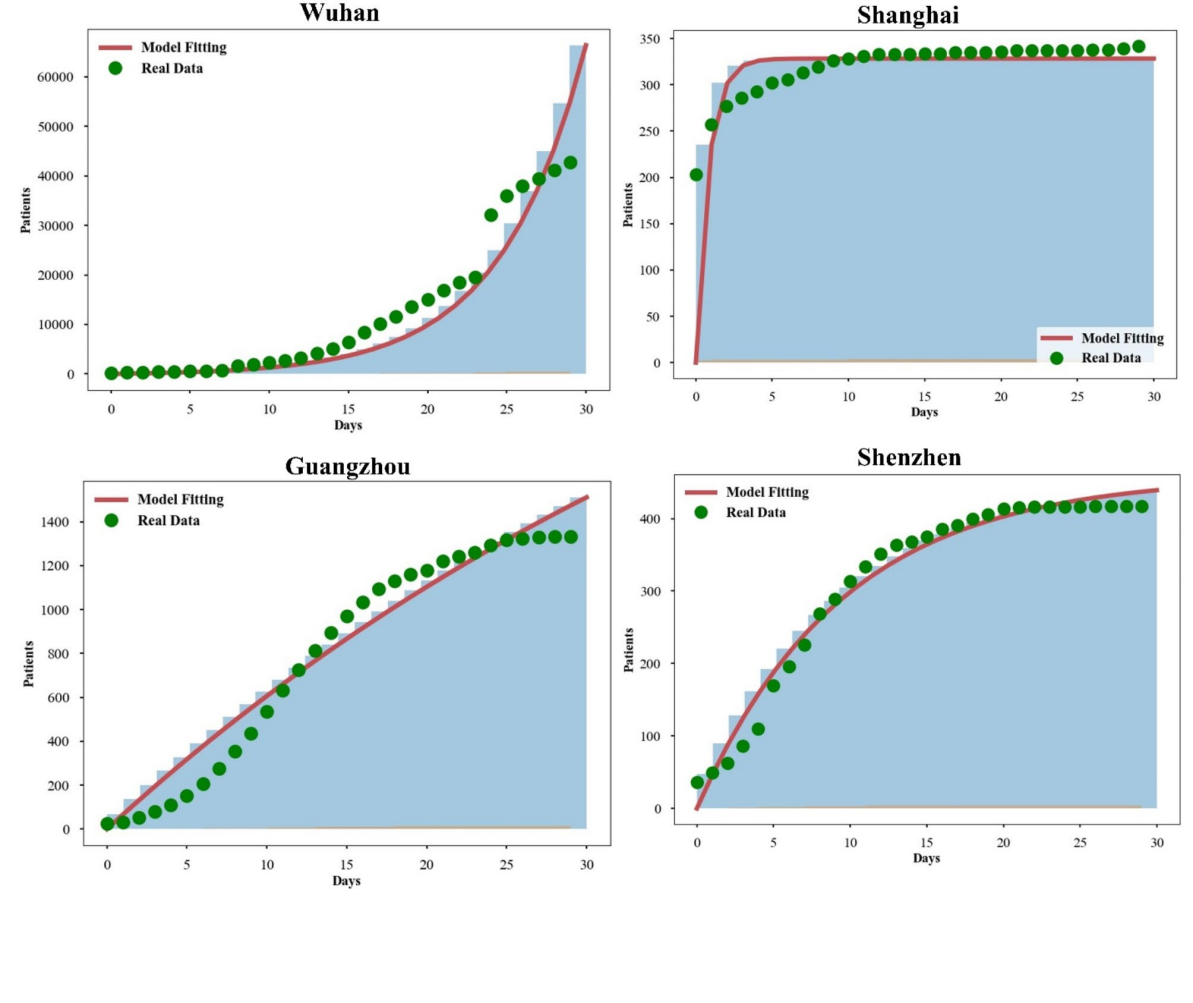
The COVID-19 data was gathered from the population of Wuhan, Shanghai, Guangzhou, and Shenzhen between July 31 and August 30, 2021, and then compared to the goodness of fit curve produced by the suggested model.

### Numerical simulation results of the SEIAPUFR model of COVID-19 in Guangzhou

The proportion of the population at each stage at any given time is determined as follows: *N* = 18,676,605, N ^∗^ = 894, E(0) = 1, A(0) = 4, P (0) = 1, U (0) = 0, I(0) = 894, F (0) = 1, R(0) = 732, S = N − I − R − E − A − F − U, C0 = 0, C1 = 0.96, C2 = 0.96, C3 = 0.95, η = 0.001, λ = 0.97, σ = 0.4, α = 0.6, ε = 1- µ, β = 0.35, γ = 1/7, µ = 0.6, ρ = 0.8, and k = 469,917,τ = 0.7,g = 23, which were reported by^44^).

From the above parameters, analyzing the time evolution presented in Fig. 11, we observe the similar results when *N* = 18,676,605. The susceptible population decreases as the recovery population increases over time until a stationary value achieved when the disease spread ends. The exposed, the infected, the asymptomatic, the respiratory specific model, non-respiratory specific model, and supervised sampling in infected populations increase until a maximum value and then they decrease and assume a null value in the end of the epidemics. Conversely, Fig. 11 shows the epidemic evolution that would have been predicted by the model for the COVID-19 outbreak, after 21 days. This suggests that social separation protocols and handling of cases, along with its severe socioeconomic repercussions, might have to persist for an extended duration.

The Fig. 12A-H shows that, with even stronger use of face masks with vaccine efficacy and perfect test accuracy, β ∈ {0.35, 0.44, 0.54, 0.63} and γ = 0.0071. Over a 120-day horizon, as shown in Fig. 12C, the model predicts an evolution of the situation that leads to a peak in the number of concurrently infected individuals around days 30, 26, 22, 19, amounting to 13.38%, 12.85%, 12.31%, 11.78% of the population for β ∈ {0.35, 0.44, 0.54, 0.63} and γ = 0.0071, respectively. Eventually, 29.44%, 28.91%, 28.37%, 27.84%of the population have respiratory specific with the virus and 20.88%, 20.34%, 19.81%, 19.28% of the population have non respiratory specific with the virus. The fraction of patients in need of supervision sampling, as shown in Fig. 12G, reach their peaks on days 30, 26, 22, 19, amounting to 40.15%, 39.62%, 39.08%, 37.48% of the population, result of an expanded testing initiative that detected a greater number of moderately symptomatic infected populations.

**Fig. 15:**
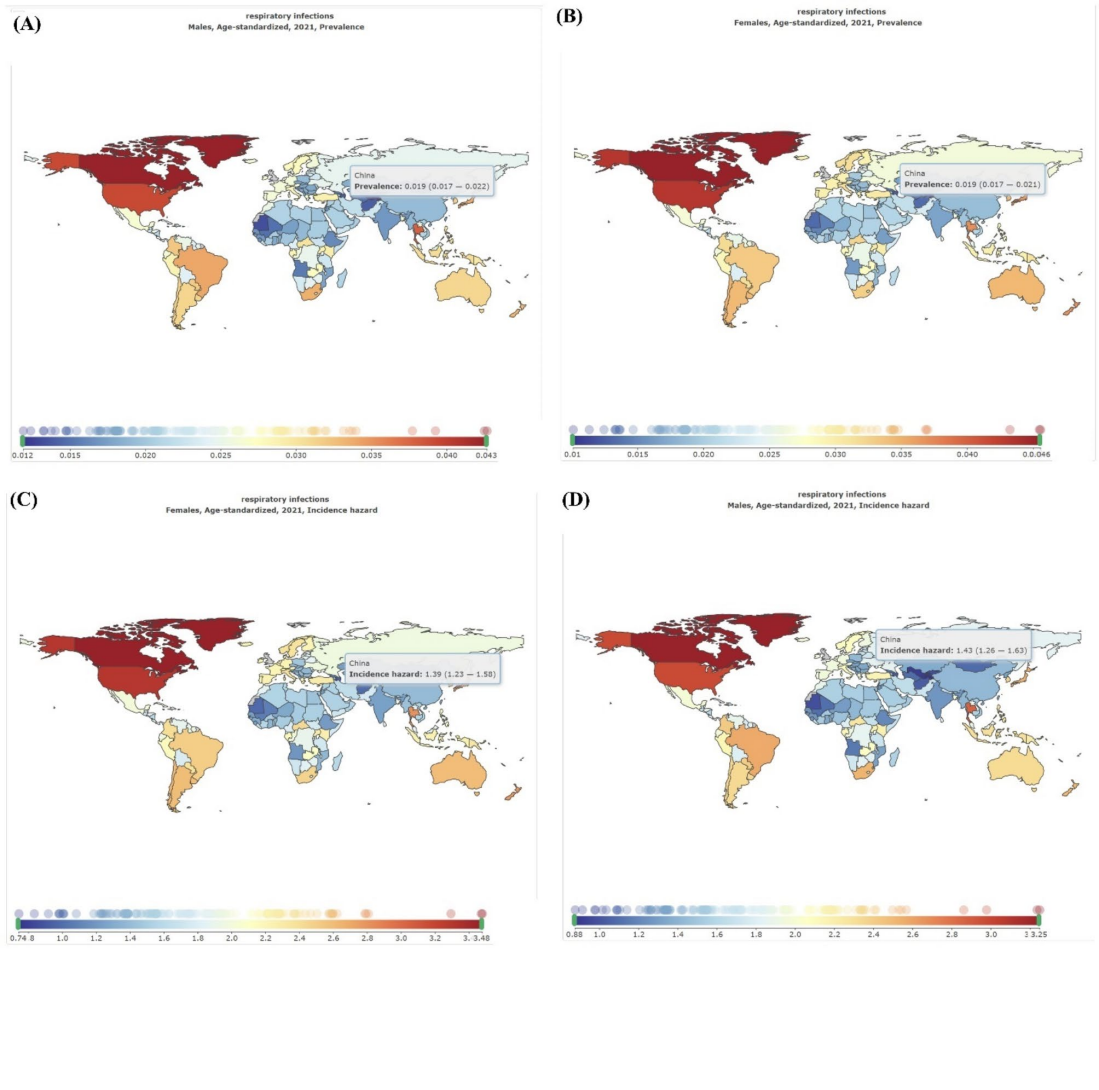
(**A**,**B**) The prevalence and incidence hazard of respiratory infectious in China form Global Burden of Disease(GBD) database in 2021 for Males and Females.

The Fig. 13A shows how the different between sub-populations of exposed individuals and respiratory specific individuals evolve over time, and it is interesting to notice that the respiratory specific sub-populations increased with exposed sub-populations at a different time and started to decrease around a point (1, 780, 000; 2, 800, 000), this suggests that, from this point on, various efforts are being taken to flatten the transmission curve, such as the use of face masks, social distance, the deployment of an effective vaccine, various degrees of testing, and the development of herd immunity. In particular, if the respiratory sub- populations are early controlled the transmission could be reduced over the time. The Fig. 13B, C shows the numerical simulation of outbreak factor in periods 5 and 7. In periods 5 and 7, the respiratory specific control, countermeasure, and perfect test accuracy rules can slow the spread of pandemic before the first peak(10 day), and latter around 115 days. In both cases(periods 5 and 7), the greater values of the epidemic are localized around t = 10, 115 days. Based on this observation, we may assume that with less control (t < 20), the infected curve has a larger peak and, after it, the spread cases for t = 10, 115, suggesting that the density of infected individuals is higher but the infection ends sooner, even when control measures are loosened. Moreover, if no reasonable control measures are put in place, an exponential viral peak will continuously occur over time.

The Fig. 13D show the number of infectious individuals during the transmission of the pandemic based on the different values of β, and γ. When a higher control is applied( β < 0.06), we see an extension of the black region, in Fig. 13D. Following the color scale, this scenario represents some peaks separated by a section of lowers values of I, which characterizes a scenario of a wave of infections^45^). Furthermore, in Fig. 13D, the color scale indicates the magnitude of I. The colored regions display combinations of β and γ for which the epidemic does not end over time. Meanwhile, for the yellow region, we observe situations related to the extinction of the epidemic, even with the relaxation of control measures and the reinfection. The higher values of I are present for higher values of β. As we see, even for a higher value of γ = 0.08, the value of infection I = 0.0. As the value of β increases, the value of infection I decreases for all values of γ. The black region, for lower values of β and higher values of γ can be explained by the fact that, with lower control measures (lower values of γ), a higher number of individuals are infected in the beginning of the spread, then, when the individuals become susceptible again, there are not more infected individuals to perpetuate the disease spread in the exposed compartment.

These scenarios, despite being surpassed, are critical in demonstrating that increased use of face masks in conjunction with vaccine efficacy and perfect test accuracy were an appropriate policy, given that the epidemic could still have had tragic outcomes in the absence of respiratory specific and supervision sampling individuals. They also propose that mandating stricter usage of face masks with vaccination effectiveness and flawless test accuracy as early as feasible, for nations still in the midst of an outbreak, leads to tremendous gains over a delayed intervention.

In epidemic transmission models, it is essential for the basic reproduction number, R0, to be smaller than one for effective pandemic control. Nonetheless, this criterion may not consistently enough for the backward bifurcation phenomenon, wherein a stable endemic equilibrium coexists with a stable disease-free equilibrium for R0 < 1. This phenomena has substantial public health implications, since it renders the standard criterion of the fundamental reproduction number being less than one necessary, yet insufficient for disease eradication. The accompanying bifurcation graph and the duration sequence corresponding to the force of infection G = βI/N are illustrated in the supplementary material Figure using the parameters *N* = 17,560,000, E(0) = 1, A(0) = 1, P (0) = 0, U (0) = 1, I(0) = 1384, F (0) = 1, R(0) = 1369, S = N − I − R − E − A − F − U, β = 0.25, γ = 0.3 for Shenzhen, *N* = 24,870,895, E(0) = 1, A(0) = 5396, P (0) = 0, U (0) = 1, I(0) = 59,342, F (0) = 1, R(0) = 45,873, S = N − I − R − E − A − F − U,β = 0.31, γ = 0.8 for Shanghai, *N* = 12,326,518, E(0) = 1, A(0) = 0, P (0) = 1, U (0) = 0, I(0) = 50,423, F (0) = 1, R(0) = 46,551, S = N − I − R − E − A − F − U,β = 0.03, γ = 1/7 for Wuhan, and Guangzhou *N* = 18,676,605, E(0) = 1, A(0) = 4, P (0) = 1, U (0) = 0, I(0) = 894, F (0) = 1, R(0) = 732, S = N − I − R − E − A − F − U, β = 0.1, γ = 1/7, displaying the disease-free equilibrium (DFE) and two endemic equilibria, where the greatest infectious equilibrium is stable and the smallest endemic equilibrium is unstable. The findings shown in this figure indicate that the local asymptotic stability of DEF and EE is contingent upon the starting sizes of the subpopulations, meaning that stable DEF coexists with stable EE. This work suggests that the standard epidemiological criterion of ensuring the fundamental reproduction number (R) of our model is less than one is not anymore sufficient, however it remains required, for successfully monitoring the propagation of an epidemic in a region.

### Model fitting

To fit the system (1), we used real data on the cumulative number of COVID-19 cases in Wuhan, Shanghai, Guangzhou, and Shenzhen, including those who were infected, and eliminated. The data was obtained from http://www.nhc.gov.cn/xcs/yqtb/list_gzbd.shtml. Specifically, we selected data that was reported over a 30-day period of the pandemic, spanning from july 31 and August 30, 2021. To get the numbers for η = 6.477e + 3, we utilized the current life expectancy in China (77.3 years). And assume that, N ^∗^ = 894, E(0) = 1, A(0) = 4, P (0) = 1, U (0) = 0, I(0) = 26, F (0) = 1, R(0) = 1, S = N − I − R − E − A − F − U, C0 = 0, C1 = 0.96, C2 = 0.96, C3 = 0.95, λ = 0.97, σ = 1/5.1^48^), α = 0.5995^48^), ε = 1- µ, β = 0.35(fitted), γ = 1/7^39^), µ = 0.6(fitted), ρ = 0.2169(fitted), and k = 469,917,τ = 0.8,g = 23, which were reported by^44^).

By using a least squares methodology, we successfully fit the model to the data and determine the parameters. Figure 14 displays empirical data on the number of infected patients, along with the fitting of a model to the number of infected symptomatic cases. It is evident from this illustration that the model accurately represents the data on the quantity of symptomatic instances of infection. All persons who are infected with COVID-19, whether they show symptoms or not, as well as those who are suspected of having the virus, are being admitted to hospitals in Wuhan, Shanghai, Guangzhou, and Shenzhen. Moreover, from Fig. 15, we observed that the prevalence of Males and Females was 0.019(0.017–0.022), and 0.019(0.017–0.021), why the incidence hazard of Males and Females was 1.39(1.23–1.58), and 1.43(1.26–1.63), respectively. The incidence hazard for respiratory infectious of Females was higher than incidence hazard for respiratory infectious of Males. Authorities can diminish the total amount of susceptible persons by suitable measures, and when accompanied by the isolation of infected people, this will lower the highest rate of transmissions. Upon completion, this effectively reduces the duration of the virus’s effects on population. Consequently, the second assertion lacks scientific foundation and is erroneous. It is crucial to diminish the highest level of disease, as this will minimize the time of necessary harsh measures(develop efficient vaccine ), contrary to the assertions made by the press and authorities that it would prolong the duration.

### Model performance

The COVID-19 predictive validity of the models reported here was tested utilizing different population sizes. Our SEIAPUFR model has consistently delivered some of the most accurate projections when compared to other models^49^). For a clear evaluation of our model performance, we have added additional sets of model and supervision samples. Moreover, the difference between our model and the traditional one is that our system can help clinicians to get the result of the nucleic acid testing based on the supervised sampling in a few minutes, while the traditional method uses the whole population for the nucleic acid testing on respiratory samples, which needs more time, and energies. We stress that these are projections of possible futures that are susceptible to a variety of model assumptions and data variability. Conducting an inadequate number of testing underestimates the probability of transmission and hinders the development of an effective vaccine; our mathematical framework can forecast the long-term consequences of misdiagnosis.

## Discussion and conclusion

SARS-CoV-2 has been difficult to control since the start of the pandemic, and the spread of multiple variants has only exacerbated this challenge, and many mathematics models have been developed to predict the transmission of COVID-19^50–52^. It has been used that control programs are developed to interrupt the chains of transmission within communities by scaling up testing, contact tracing, and isolation since the early days, but heavy losses have also been paid, both politically and economically^53–55^. Since SARS-CoV-2 can spread from people who are pre-symptomatic, symptomatic, or essentially asymptomatic, diagnosis and isolation based on symptoms alone will not be enough to stop the virus from spreading^56,57^. This group of persons is likely to become populationwide covert transmitters. The estimations of SARS-CoV-2 transmissibility, which mainly rely on observed counts of cumulative infections, might be influenced by restricted and biased testing^58^. These tests are time-consuming and cannot be performed on all of the population, and substantial false negative rates have also been reported^59^. As a result, using effective targeted control strategies to detect infected individuals might be one way to flatten transmission curves and enable the resumption of social activities and economic recovery. In this study, we combined a vaccine method, a perfect test accuracy, a respiratory specific model, and disease supervision sampling into a single host population for disease control to mirror those findings from studies of historical pandemic. The parameter estimation methodology was well used for determining the nearest parameter values to the actual data while producing the curve that best matches the real data^60^. The novel SEIAPUFR model also replicated accurately and extended the classic SEI(A)R propagation model, while simulating the significance of supervision sampling in control of respiratory communicable disease at different stages. These findings indicate that reasonable control measures can lower the transmission rate β to slow spread of SARS-CoV-2, resulting in a reduction in the number of people exposed and infected. If reasonable and effective interventions are adopted to suppress respiratory transmission during the early stage of the pandemic, it can flatten the transmission curve-that is, to extend the period over which cases occurred, which will slow the momentum of the outbreak to reduce medical pressure. In contrast, loosened containment can accelerate the spread, so that an exponential pandemic peak will inevitably occur over time. The number of infected people increases with an increasing β, so as the required proportion of supervision sampling. Whereas the removal rate γ tends to correlate with different levels of medical care and disease severity in the area, including non-life-threatening cases (asymptomatic and paucisymptomatic; minor and moderate infection) and potentially life-threatening cases (major and extreme infection) that require intensive care units (ICUs). Classic SEI(A)R model can be used to forecast or simulate future transmission scenarios under various assumptions about parameters governing transmission, disease, and immunity, while long-term simulation cannot be done with constant parameters^61^. This is due to the fact that the transmission rate β and the removed rate γ must be modified over time based on the adoption of control measures^62^. The original versions of the controversial model from the Institute for Health Metrics and Evaluation (IHME) fell into this category. The disease-specific parameters driving model can be modified to test how the pandemic may change under various assumptions about the disease and implementation of control measures. In our study, we assume that the β value will be different in different scenarios based on the implementation of control measures (such as lockdown, social distancing, hygiene advice and use of masks) and vaccine effectiveness. The model predicts an evolution that is concurrently the peaks of population (including exposed, asymptomatic, respiratory specific, non-respiratory specific, supervision sampling) occur earlier, faster, and higher as the β value increases. Such excessively rapid peaks can easily overburden the healthcare system and compress the time to improve clinical care strategies and capacity. This phenomenon may explain why the Chinese government was so aggressive in taking control measures early in the pandemic^63^. Absolutely, these containment measures are very useful, mainly in that the exposure sub-populations increase with the respiratory-specific sub-populations early in the pandemic, and then start to decrease after a certain inflection point. It means that control measures during the pandemic have interrupted the chain of transmission-mainly by reducing respiratory spread, reflected in sharply reduced exposures owing to fewer respiratory-specific populations, which is forward to flatten the transmission curve. Hence, we firmly believe it is logical to distinguish between respiratory and non-respiratory transmission based on the disease characteristics of COVID-19, just like other modified SEI(A)R models such as SEIRS, SVEIR and SEPIAR to more accurately describe transmission for different modeling purposes. Identification of people in the community who are infected is an integral element of outbreak management. Some countries monitor COVID-19 fluctuations with surveys of virus prevalence in the wider population, but these have mostly been one-off activities due to high costs. Therefore, real-time supervision sampling survey based on population samples is a crucial component of effective targeted control strategies. Whether in targeted capture of real-time viral spread, or horizontal and longitudinal designs for prevalence and relapse tracking, this supervision sampling has significant advantages as SARS-CoV-2 persists. The Real-time Assessment of Community Transmission-1 (REACT-1) study^64^, a representative community-wide sampling survey that is tracking prevalence of SARS-CoV-2 across England through repeated random population-based sampling. Other studies such as Zambia’s cluster sample survey for estimated numbers of SARS-CoV-2 infections^65^, and the Office for National Statistics (ONS) Coronavirus Infection Survey^66^, through repeated cross-sectional household surveys with additional serial sampling and longitudinal follow-up, presented the spread trends of COVID-19 in England. The concepts of supervision sampling are well used in these studies to track virus and estimate prevalence. However, these surveys are also unilateral and imperfect to a certain extent. On the one hand, most sample surveys are tended to maximize randomness and representativeness to objectively reflect the pandemic characteristics. In fact, there are still many circumstances where we need targeted routine asymptomatic testing for high-risk groups and areas to prevent outbreaks in at-risk settings. On the other hand, as the survey progresses, the sampling proportions should be different among communities due to adoption of control measures. Even the targeted proportions should also be different due to differences in transmission capacity, according to the contemporaneous numbers of asymptomatic, respiratory symptoms, and non-respiratory symptoms. This program is designed to reflect the objective regulation of the pandemic from multiple levels by combining multiple groups of people in the same period. We adjust the proportion of supervision sampling based on the number of susceptible and exposed individuals, as well as differences in transmission rate. It is not difficult for us to find that the tendency in propagation curve of supervision sampling is close to respiratory specific model. This may be explained that respiratory infection is the main way of COVID-19 spread, which hence determines the number of exposed people to a certain extent. While the proportion of peak sampling is proportional to the β value, and both decrease with the adoption of control measures. This means that sampling in low-prevalence areas should be appropriately reduced, where supervision sampling should be only aimed at preventing a resurgence of SARS-CoV-2 transmission. In a rapidly evolving epidemic, during which ongoing targeted surveillance is essential to guide public health response^67^. Regarding diagnostic tests for COVID-19, current conventional molecular techniques for detecting SARS-CoV-2 in respiratory samples utilize non-specific real-time polymerase chain reaction with reverse transcription strategies, targeting RNA-dependent RNA polymerase and E genes. The tests were labor-intensive and are unable to be administered to all vulnerable persons within the community; elevated rates of false negatives have been identified, and accredited institutions equipped with costly apparatus are required. There is an urgent need for quick tests exhibiting high sensitivity and specificity that may be readily implemented in real-world environments such as hospitals, schools, airports, and train stations. Certain laboratories are advancing towards the development of a 15-minute test for the simultaneous detection of SARS-CoV-2 immunoglobulins IgM and IgG in human blood. In contrast, surveillance sampling control of this respiratory infectious disease may have prospective value relative to routine surveillance, especially virus testing in the wider population. For one thing, this program could more accurately describe the natural laws of COVID-19, and data from supervision sampling or similar surveys could be used to target local public health or vaccination campaigns, which would facilitate policy response and timely implementation of public health interventions^68^. For another, the information from community-based surveillance can be used to calibrate and merge other data streams, not only symptomatic testing but also the use of mobility data and sewage-based sampling of viral RNA^69^. Additionally, regardless of low-prevalence or disease control, the targeted sampling can help to provide early warning of any upturn in infections. With the persistence of SARS-CoV-2, this targeted surveillance will hopefully become the main policy to contain respiratory infectious diseases in the future. Nonetheless, this junior mathematical model had several limitations. First, although the typical cities for the COVID-19 outbreak are the four districts selected for the simulation, the generalisability of the findings to all districts of China is unknown. Second, it is not clear whether such transmission simulations based on urban populations are applicable to rural areas due to large differences in population density and medical care. Third, the number of individuals selected for the model simulation stems from the national government work report, including the total population, the infected, the removed, vaccination coverage, etc. These values may deviate from the actual results to a certain extent, although we have tried our best to avoid. Finally, we did not take into account the effects of specific conditions, such as containment measures adjustment, variant emergence, increased social mixing, and population migration, which may be seriously addressed in our future.

In conclusion, we employ a least squares method to enhance our comprehension of contact-based virus transmission. The framework of independent variables as infected carriers in two dimensions is comprehensible and demonstrates potential for enhancing the comprehension of infectious illness epidemics. It is easier to access than different designs and can capture random interactions overlooked by traditional methods. The simulations demonstrate a reasonably good prediction of infection paths, including primary as well as secondary waves, for COVID-19 information collected from four nations with significantly varied characteristics.

## Data Availability

The datasets generated and/or analysed during the current study are available in the http://www.nhc.gov.cn/xcs/yqtb/list_gzbd.shtml, https://www.geodata.cn/sari2020/web/index.html, and VizHub - GBD Results (healthdata.org).
